# Helminth extracellular vesicles co‐opt host monocytes to drive T cell anergy

**DOI:** 10.1002/jev2.70027

**Published:** 2025-01-16

**Authors:** Anne Borup, Mohammad Farouq Sharifpour, Litten S. Rossen, Bradley Whitehead, Anders T. Boysen, Rikke Olesen, Anja B. Bohn, Andrea Ridolfi, Marco Brucale, Francesco Valle, Lucia Paolini, Annalisa Radeghieri, Paolo Bergese, Kim Miles, Margaret Veitch, Tamara Thomas, Roland Ruscher, Phurpa Wangchuk, Paul Giacomin, Alex Loukas, Peter Nejsum

**Affiliations:** ^1^ Department of Clinical Medicine Aarhus University Aarhus Denmark; ^2^ Department of Infectious Diseases Aarhus University Hospital Aarhus Denmark; ^3^ Australian Institute of Tropical Health and Medicine James Cook University Cairns Queensland Australia; ^4^ Department of Biomedicine, FACS Core Facility Aarhus University Aarhus Denmark; ^5^ Department of Physics and Astronomy and LaserLaB Amsterdam Vrije Universiteit Amsterdam Amsterdam The Netherlands; ^6^ Consorzio Interuniversitario per lo Sviluppo dei Sistemi a Grande Interfase (CSGI) University of Florence Florence Italy; ^7^ Consiglio Nazionale delle Ricerche (CNR) Istituto per lo Studio dei Materiali Nanostrutturati (ISMN) University of Bologna Bologna Italy; ^8^ Department of Molecular and Translational Medicine University of Brescia Brescia Italy; ^9^ Consiglio Nazionale delle Ricerche (CNR), Institute for Research and Biomedical Innovation (IRIB) University of Palermo Palermo Italy

**Keywords:** Ascaris, colitis, extracellular vesicles, helminth, host‐parasite interaction, immune modulation, inflammatory bowel disease, monocytes

## Abstract

Parasitic helminths secrete extracellular vesicles (EVs) into their host tissues to modulate immune responses, but the underlying mechanisms are poorly understood. We demonstrate that *Ascaris* EVs are efficiently internalised by monocytes in human peripheral blood mononuclear cells and increase the percentage of classical monocytes. Furthermore, EV treatment of monocytes induced a novel anti‐inflammatory phenotype characterised by CD14^+^, CD16^−^, CC chemokine receptor 2 (CCR2^−^) and programmed death‐ligand 1 (PD‐L1)^+^ cells. In addition, *Ascaris* EVs induced T cell anergy in a monocyte‐dependent mechanism. Targeting professional phagocytes to induce both direct and indirect pathways of immune modulation presents a highly novel and efficient mechanism of EV‐mediated host‐parasite communication. Intra‐peritoneal administration of EVs induced protection against gut inflammation in the dextran sodium sulphate model of colitis in mice. *Ascaris* EVs were shown to affect circulating immune cells and protect against gut inflammation; this highlights their potential as a subject for further investigation in inflammatory conditions driven by dysregulated immune responses. However, their clinical translation would require further studies and careful consideration of ethical implications.

## 1 INTRODUCTION

Soil‐transmitted helminths (STHs) are a major cause of neglected tropical diseases, with 1.5 billion people estimated to be infected worldwide (WHO, 2022). Although infection risk is associated with inadequate sanitation and hygiene in developing countries, the high prevalence is also attributed to the ability of helminths to develop a successful, resilient chronic infection combined with their high reproductive output (Loukas et al., 2021). Helminth infection is associated with modulation of the host immune response with the downregulation of the pro‐inflammatory type 1 and type 17 responses in favour of a modified anti‐inflammatory type 2 response (Zakeri et al., 2018). Helminths potently skew the immune response by promoting host IL‐10 and TGF‐β production, and expanding alternatively activated macrophages, regulatory T cells (Tregs) and eosinophils (Ryan et al., 2020). The expansion of T regs and T cell anergy is conserved across helminths and, therefore, may represent a key mechanism of host‐parasite communication (White et al., 2020). The ability to suppress pro‐inflammatory responses has prompted researchers to consider helminths and their secreted products as a source of novel therapeutic modalities to mitigate autoimmune or inflammatory diseases, such as inflammatory bowel disease (IBD) and rheumatoid arthritis (Smallwood et al., 2017). This potential has been supported by several studies showing that helminth therapy alleviates the severity and clinical symptoms of autoimmune diseases (Smallwood et al., 2017; White et al., 2020).

In recent years, helminths have been shown to release extracellular vesicles (EVs) as part of their excretory‐secretory products (ESPs) (Drurey & Maizels, 2021; Mardahl et al., 2019). EVs are important mediators of communication between cells and may, therefore, be crucial for host‐parasite interaction and infection biology of the pathogens. EVs are nanoscale (< 1000 nm) particles enclosed with a phospholipid bilayer membrane and can carry a diverse cargo of bioactive proteins, nucleic acids, and lipids (Van Niel et al., 2018). The complex composition of EVs makes them ideal mediators of communication in various physiological and pathological processes. Despite some selected studies indicating that helminths utilise EVs to evade and suppress the host's immune response, their roles in host‐parasite interaction remain poorly understood (Drurey & Maizels, 2021).

In this study we sought to expand the knowledge of the role of EVs in helminth‐induced immunomodulation by investigating the nematode *Ascaris suum*. This porcine roundworm serves as a faithful model for its human counterpart, *Ascaris lumbricoides*, the most prevalent STH in humans (Else et al., 2020). Several studies have shown that *Ascaris* spp. downregulate pro‐inflammatory cytokines and chemokines whilst increasing the expression of type 2 cytokines such as interleukin‐4 (IL‐4) and transforming growth factor‐β (TGF‐β) (Midttun et al., 2018; Nogueira et al., 2016; Silva et al., 2021). We have previously demonstrated that *A. suum* releases immunomodulatory EVs as part of its ESP complement (Borup et al., 2022). Herein, we examined the internalisation and functional role of *A. suum* EVs in human peripheral blood mononuclear cells (PBMCs). We showed that *A. suum* EVs target human monocytes to generate an anti‐inflammatory phenotype that mediates T cell anergy, and that administration of *A. suum* EVs to mice protected against chemically induced colitis. Our findings provide an important insight into how EVs from an intestinal helminth may manipulate circulating immune cells to downregulate the systemic immune response.

## 2 MATERIALS AND METHODS

### 2.1 Helminth culture

Ex vivo cultures of adult *A. suum* were performed as previously described (Borup et al., 2022). Briefly, adult *A. suum* worms were recovered from the local abattoir (DAT‐Schaub A/S, Herning, Denmark) and pre‐washed in 37°C saline (0.9% NaCl (Sigma‐Aldrich, St. Louis, MO, USA)). The worms were allocated to T175 flasks in a ratio of 3:2 females to males per flask. The worms were washed in saline (0.9% NaCl) containing antibiotics and antimycotics (200 µg/mL Streptomycin, 200 U/mL Penicillin G, 1.25 µg/mL Amphotericin B from *Streptomyces* sp. and 10 µg/mL Ciprofloxacin) (all reagents from Sigma‐Aldrich, St. Louis, MO, USA). The washing procedure consisted of four incubation periods of 15 min and three incubation periods of 1 h at 37°C. Thereafter, worms were incubated at 37°C and 5% CO_2_ for 3 days in 175 mL RPMI‐1640 + antibiotic‐antimycotic mixture (100 µg/mL Streptomycin, 100 U/mL Penicillin G, 0.63 µg/mL Amphotericin B and 5 µg/mL Ciprofloxacin). The conditioned media were collected and exchanged every 24 h. To check for bacterial and mycoplasma contamination, the conditioned media was analysed using Luria Bertani agar plates and a MycoAlert Mycoplasma Detection kit (Lonza Group AG, Gampel, Switzerland). The conditioned media was pre‐cleared for eggs and debris by centrifugation at 300 ×g for 10 min and 2000 ×g for 10 min. The supernatant was kept at −80°C until further processing.

### 2.2 In‐vivo labelling of *A. suum* EV

In‐vivo labelling of *A. suum* EVs was based on a validated protocol using the related ascarid *Anisakis* spp. by Boysen et al., 2020. Worms were distributed, washed, and incubated for cleaning as described above. Afterwards, the worms were incubated for 24 h with RPMI‐1640 with an antibiotic‐antimycotic mixture (100 µg/mL Streptomycin, 100 U/mL Penicillin G, 0.63 µg/mL Amphotericin B and 5 µg/mL Ciprofloxacin) and 1 µM 1,2‐dioleoyl‐sn‐glycero‐3‐phosphoethanolamine‐N‐lissamine rhodamine B sulfonyl (DOPE‐Rho) (Avanti Polar Lipids, Alabaster, AL, USA). The conditioned media was discarded to remove excess dye, and a fresh RPMI‐1640 + antibiotic‐antimycotic mixture was added for an additional 48 h of incubation with daily collection of conditioned media. After collection, the conditioned media was checked for bacterial contamination as described above. The conditioned media were pre‐cleared for eggs and debris by centrifugation at 300 ×g for 10 min and 2000 ×g for 10 min. The supernatant was kept at −80°C until further processing.

### 2.3 Separation and characterisation of EV

A detailed characterisation of *A. suum* EVs obtained by the used separation method has been described by Borup et al., 2022.

For unlabelled samples, conditioned media from three days of culture were pooled and concentrated from 270 mL to 500 µL using an Amicon Ultra‐15 Centrifugal Filter Unit 10 kDa cut‐off (Merck Life Science, Burlington, MA, USA). For DOPE‐Rho labelled samples, conditioned media from two days of culture were pooled, and the same Amicon filters were used to concentrate to 500 µL. Afterwards, EVs were separated by size exclusion chromatography (SEC) using qEVoriginal/75 nm columns (iZON Science Ltd, Christchurch, New Zealand) as previously described (Borup et al., 2022). Fractions of 500 µL were collected, and fractions 7–10 contained EVs, fractions 11–15 were a mixture of EVs and proteins, and fractions 16–25 included proteins. The last fractions were used as EV‐depleted ESP for control in functional assays. The in‐vivo labelled EVs are termed DOPE‐Rho EVs in the following sections.

Nanoparticle tracking analysis (NTA) was used to determine the concentration and sizes of particles using the Nanosight NS300 system (Malvern Panalytical, Malvern, UK) coupled with a NanoSight syringe pump (Malvern Panalytical, Malvern, UK). The settings corresponded to the previous description (Zendrini et al., 2020). The samples were infused into the system by a continuous flow of 20 µL/min and videos of 5 × 60 sec were recorded. Particles were detected at a camera level of 15. Processing of data was conducted using the Nanosight 3.4 software (Malvern Panalytical, Malvern, UK) applying a detection threshold of five.

Protein concentration was measured by the Pierce™ BCA Protein Assay kit (Thermo Fisher Scientific, Waltham, MA, USA) according to the manufacturer's protocol using 10 µL of the sample in a 96‐well plate.

### 2.4 Cryogenic transmission electron microscopy (Cryo‐TEM)

Samples used for cryo‐TEM had a maximum of one freeze‐thaw cycle after enrichment. After thawing, samples were spun at 2,000 ×g on a one‐speed tabletop centrifuge for 5 min to remove aggregates introduced by the freeze‐thaw cycle. Immediately after this clearing spin, 4 µL was dispersed to a glow‐discharged lacey formvar carbon‐coated copper grid (Ted Pella Inc., Redding, CA, USA) and blotted on filter paper. The grids were then plunged into liquid ethane (approximately −183°C). Vitrified samples were thereafter stored in liquid nitrogen before imaging. A Fischione Model 2550 cryo transfer tomography holder was used to transfer the samples into the electron microscope. A JEM 2200FS (JEOL, Tokyo, Japan) cryo‐TEM with an in‐column energy filter (Omega filter) allowing for zero‐loss imaging was used to image the samples. The acceleration voltage was 200 kV, and under low dose conditions with a 25 eV slit in place, energy‐filtered images were digitally recorded with a TVIPS F416 camera. Images were subsequently processed, and the size of the lipid core of individual EVs was measured in ImageJ 1.52a (NIH) (Schneider et al., 2012).

We have submitted all relevant data of our experiments to the EV‐TRACK knowledgebase (EV‐TRACK ID: EV240187) (Van Deun et al., 2017).

### 2.5 PNGase treatment of EV

EVs separated by SEC were treated with 400 U/mL PNGase F in PBS (Thermo Fisher Scientific, Waltham, MA, USA) by incubation for 24 h at 37°C. Subsequently, the sample was centrifuged at 110,000 ×g for 65 min at 4°C to remove glycans and PNGase F residue.

### 2.6 Atomic force microscopy

The efficacy of the PNGase treatment was assessed by atomic force microscopy (AFM). To this aim, borosilicate glass coverslips (Menzel Gläser, Braunschweig, Germany) were cleaned and functionalised with poly‐L‐lysine following the procedure described in Borup et al., 2022. EV preparations were deposited on functionalised glass slides, left to adsorb for 30 min at 4°C, and then inserted in the AFM fluid cell (see below) without further rinsing. The concentration of the samples was adjusted in order to maximize the number of separated EVs on the surface. All AFM images were recorded on a Multimode 8 microscope (Bruker, USA) equipped with a Nanoscope V controller and a type JV piezo scanner. Samples were measured in a sealed fluid cell filled with ultrapure water using SNL‐A probes (Bruker, USA). Raw images were processed with Gwyddion v2.58 (Nečas & Klapetek, 2012) for background subtraction. The structural integrity of EVs before and after PNGase F treatment was assessed via AFM as described elsewhere (Ridolfi et al., 2020). The PNGase‐treated EVs (30,000 particles/cell) were used to stimulate PBMCs before the cells were analysed by flow cytometry as described below.

### 2.7 PBMC isolation

Human buffy coats from healthy, anonymous donors were obtained from the Danish blood bank at Aarhus University Hospital, Denmark. PBMCs were separated using density gradient centrifugation using SepMate‐50 tubes (STEMCELL Technologies, Vancouver, Canada) with Ficoll‐Paque PLUS (GE Healthcare, Uppsala, Sweden). Buffy coats diluted 1:1 in 2% heat‐inactivated foetal bovine serum (HI‐FBS) (Biowest, Nauillé, France) were added to the SepMate‐50 tubes prepared with 15 mL Ficoll‐Paque PLUS. The tubes were centrifuged at 1200 ×g for 10 min at RT. Supernatants were decanted into 50 mL Falcon tubes, refilled with 2% FBS, and centrifuged twice at 500 ×g for 10 min at RT. Cells were resuspended in 1 mL of 2% FBS and 20% dimethyl sulfoxide for cryopreservation.

### 2.8 Uptake of EV in PBMCs detected by flow cytometry and imaging flow cytometry

PBMCs from three donors were thawed and seeded in RPMI‐1640 with 10% HI‐FBS at a density of 1 million cells per well in a 24‐well plate for suspension cells (Sarstedt, Nümbrecht, Germany). Simultaneously with seeding, cells were stimulated with 30,000 particles of DOPE‐Rho EVs per cell (as measured by NTA) [the dose is based on previous work (Borup et al., 2022)]. Cells were incubated overnight (∼16‐18 h) at 37°C and 5% CO_2_ before being harvested and treated with human Fc Block (BD Biosciences, Franklin Lakes, NJ, USA). The cells were labelled with the following antibodies: anti‐CD3‐V450, anti‐CD4‐Alexa Flour 700, anti‐CD8‐Alexa Flour 488, anti‐CD14‐BV786 and anti‐CD19‐PE‐Cy7. Fixable Viability Stain 510 (FVS501) was used as a live/dead stain (details of the antibodies, source, and volume are shown in Table S1). All antibodies were titrated, and the minimum concentrations for the separation of positive and negative populations were chosen. Compensation was performed using PBMCs. PBS with 0.5% bovine serum albumin and 0.09% sodium azide was used as a stain buffer. Cells were stained for 30 min at RT. Apart from the stimulated cells, three controls were included: cells stimulated with 30,000 unlabelled EVs per cell (negative control for the DOPE‐Rho EVs), a negative control (cells only stained with antibodies), and a blank (no treatment and no staining). The samples were acquired on a NovoCyte Quanteon 4025 Flow Cytometer with four lasers (405 nm, 488 nm, 561 nm, and 637 nm) and 25 fluorescence detectors (Agilent Technologies, Santa Clara, CA, USA) and NovoExpress software v1.5.6 (Agilent Technologies, Santa Clara, CA, USA). Data were analysed in FlowJo™ v10.8 software (BD Biosciences, Franklin Lakes, NJ, USA). The gating strategy is provided in Figure S6‐A, S6‐B, and S6‐C.

To further assess the uptake of the DOPE‐Rho EVs, quantitative and high‐throughput imaging flow cytometry was performed. Five million PBMCs from one donor were stimulated and stained following the same protocol as above; however, the antibody panel was reduced and divided into two panels: 1) anti‐CD3‐V450, anti‐CD19‐PE‐Cy7 and FVS510; 2) anti‐CD14‐APC and FVS510 (details of the antibodies, source and concentration are shown in Table S1). Compensation was performed using anti‐mouse CompBeads (IgG) (BD Biosciences, Franklin Lakes, NJ, USA) stained with each fluorochrome‐conjugated antibody. The same controls as mentioned above were included. The cells were analysed on the Amnis ImageStream^X^ Mk II (Luminex, Austin, TX, USA) equipped with four lasers (405 nm, 488 nm, 642 nm and 785 nm (sidescatter)). Laser power for the four lasers was 120 mW, 170 mW, 150 mW and 0.50 mW, respectively. Samples were acquired using INSPIRE v200.1.681.0 (Luminex, Austin, TX, USA) at 60x magnification. All data were analysed in IDEAS v6.3 (Luminex, Austin, TX, USA). The gating strategy is provided in Figure S7‐A and S7‐B. Internalisation of EVs was determined by creating an adaptive erode 75 mask (*mask: AdaptiveErode (M01, Ch01, 75*) created based on the brightfield image and calculation of the internalisation score within that mask. Examples of tested, but not used masks can be seen in Figure S1.

### 2.9 Stimulation of PBMCs with EV for functional studies

In a six‐well plate (Sarstedt, Nümbrecht, Germany), 1.5 million PBMCs were seeded in RPMI‐1640 with 10% HI‐FBS. Immediately after seeding, the cells were stimulated with EVs using different numbers of particles per cell (based on NTA). The number of particles used in the different experiments ranged between 1,000, 3,500, 10,000, 30,000, and 100,000 particles/cell. For each EV dose, a normalised EV‐depleted control sample was included in an equal amount of protein (measured by BCA) as for the EV sample. Furthermore, a negative RPMI control was included. Cells were incubated overnight (∼16–18 h) and further stimulated or harvested for flow cytometry analysis.

### 2.10 Flow cytometry analysis for functional studies

PBMCs were washed in stain buffer consisting of PBS + 0.5% BSA + 0.09% sodium azide, treated with human Fc Block (BD Biosciences, Franklin Lakes, NJ, USA) for 15 min, and labelled for 30 min in 50 µL BD Horizon Brilliant Stain Buffer (BD Biosciences, Franklin Lakes, NJ, USA). The following antibodies were used to evaluate the monocyte surface markers: anti‐CD86‐BUV395, anti‐CD16‐BUV496, anti‐CD169‐BV421, anti‐PDL1/CD274‐BV605 (clone: MIH1), anti‐CD9‐BV786 (clone: ML13), anti‐CD33‐PerCP‐Cy5.5, anti‐HLA‐DR‐PE, anti‐CD14‐PE‐Cy7, anti‐CCR2/CD192‐APC, and anti‐CD47‐Alexa Flour 700 (details of the antibodies, source, and concentration are shown in Table S1). Live/dead fixable near‐IR (Invitrogen, Waltham, MA, USA) were used as live/dead stains. All antibodies were titrated and the minimum concentrations for separation of positive and negative populations were chosen. Compensation was performed using either anti‐mouse CompBeads (IgG) (BD Biosciences, Franklin Lakes, NJ, USA) or PBMCs stained with each fluorochrome‐conjugated antibody. The samples were washed twice with stain buffer after labelling and analysed using a LSRFortessa X‐20 Cell Analyzer equipped with five lasers (355 nm, 405 nm, 488 nm, 561 nm, and 640 nm) and 19 filters (BD Biosciences, Franklin Lakes, NJ, USA) and BD FACSDiva software v8.0.1 (BD Biosciences, Franklin Lakes, NJ, USA). Data were analysed using FlowJo™ v10.8 software (BD Life Sciences). Furthermore, the median fluorescence intensity (MFI) of programmed death‐ligand 1 (PD‐L1) and CC chemokine receptor 2 (CCR2) were calculated. The gating strategy for this experiment and the experiment using PNGase F treated EVs is provided in Figure S8.

### 2.11 Inhibition of CD16 and CCR2 cleavage

PBMCs were thawed and rested overnight in RPMI‐1640 supplemented with 10% HI‐FBS. The following day, the cells were counted, and 1.5 million cells were incubated with 5 µM or 20 µM TNF‐α processing inhibitor‐0 (TAPI‐0) (R&D Systems, Minneapolis, MN, USA) for 30 min at 37°C. The inhibitor did not affect viability. After incubation, the cells were stimulated with either RPMI‐1640, EV‐depleted sample, or 30,000 particles/cell of EVs for 30 min. Following stimulation, the cells were harvested and analysed by flow cytometry. Cells were labelled with: anti‐CD16‐BUV496 (clone: 3G6), anti‐CD33‐PerCP‐Cy5.5 (clone: WM53), anti‐CD14‐PE‐Cy7 (clone: M5E2), anti‐CCR2/CD192‐APC (clone: K036C2), and zombie violet viability dye (Biolegend, USA) (details of the antibodies, source, and concentration are shown in Table S1) and analysed using an LSRFortessa X‐20 Cell Analyzer (BD Biosciences, Franklin Lakes, NJ, USA) with BD FACSDiva software v8.0.1 (BD Biosciences, Franklin Lakes, NJ, USA). All antibodies were titrated and the minimum concentrations for separation of positive and negative populations were chosen. Compensation was adjusted using anti‐mouse CompBeads (IgG) (BD Biosciences, Franklin Lakes, NJ, USA) or PBMCs stained with each fluorochrome‐conjugated antibody. Data were analysed using FlowJo™ v10.8 Software (BD Biosciences, Franklin Lakes, NJ, USA). Furthermore, the MFI of CD16 and CCR2 was calculated. The gating strategy is provided in Figure S9.

### 2.12 T cell activation assay

For antibody stimulation of PBMCs, a 96‐well plate was coated with either 1 µg/mL anti‐CD3 (clone: OKT3) and 1 µg/mL anti‐CD28 (clone: CD28.2) or 1 µg/mL mouse IgG1 isotype (clone: P3.6.2.8.1) and 1 µg/mL mouse IgG2a isotype (clone: eBM2a) at 4°C overnight (all reagents from ebioscience, San Diego, CA, USA). The isotypes were controlled for non‐specific Fc‐binding. As described in section 2.7, human PBMCs were stimulated with EVs (1,000, 3,500, or 10,000 particles/cell) or EV‐depleted ESP samples normalised to protein concentration immediately during seeding in a 96‐well plate. After 16 h, the total cell suspension was transferred to the anti‐CD3/anti‐CD28 solution or isotype control coated plate and incubated at 37°C for 24 or 48 h. To evaluate viability, 30 µL of the cell culture was analysed using a NucleoCounter NC‐202 (Chemometec, Allerød, Denmark) with Via2‐Cassettes (Chemometec, Allerød, Denmark) according to the manufacturer's instructions. Live and dead cells were discriminated by acridine orange and 4′,6‐diamidino‐2‐phenylindole (DAPI) staining. The cell supernatant was harvested by transferring the supernatant to a 96‐well u‐shaped plate. The plate was centrifuged at 500 ×g for 5 min. The concentrations of selected cytokines were analysed as described in section 2.12.

### 2.13 Monocyte depletion before T cell activity assay

CD14^+^ cells were depleted before conducting the T cell activity assay described in section 2.10. Monocytes were depleted from PBMCs by positive selection using CD14 microbeads (Miltenyi Biotec, Bergisch Gladbach, Germany) and LS columns (Miltenyi Biotec, Bergisch Gladbach, Germany) according to the manufacturer's protocol. To evaluate the efficacy of the depletion of CD14^+^ cells, 1 million cells from before and after the depletion were stained with anti‐CD14‐PE‐Cy7 (clone: M5E2, BD Biosciences, Franklin Lakes, NJ, USA) and analysed using an LSRFortessa X‐20 Cell Analyzer (BD Biosciences, Franklin Lakes, NJ, USA). Of the cells, 97.8% were CD14^−^. The same donors were used as a control for the monocyte‐depleted population during antibody stimulation. Following isolation, the cells were counted and stimulated with EVs (3,500 or 10,000 particles/cell), as described in section 2.7. After 16 h, the total cell suspension was transferred to the anti‐CD3/anti‐CD28 or isotype control coated plate and incubated at 37°C for 24 or 48 h. The cell viability was determined by adding 0.01 mg/mL resazurin (Biotium Inc., Fremont, CA, USA) to the cells, and the fluorescence intensity was measured at 563 nm using a FLUOstar Omega microplate reader (BMG Labtech, Ortenberg, Germany). The cell supernatant was harvested by transferring the supernatant to a 96‐well u‐shaped plate and centrifuging it at 500 ×g for 5 min. The concentrations of selected cytokines were analysed as described in section 2.12.

### 2.14 Quantification of cytokines with ELISA

After T cell activity assays, cell supernatants were analysed for levels of human IL‐10, IL‐2, and interferon‐γ (IFN‐γ) using DuoSet ELISA kits (all are from R&D Systems, Minneapolis, MN, USA) according to the manufacturer's protocol using Nunc MaxiSorp™ flat‐bottom plates (Thermo Fisher Scientific, MA, USA). Plates were developed using streptavidin‐HRP (R&D Systems, Minneapolis, MN, USA) and TMB X‐tra (Kementec Solutions A/S, Taastrup, Denmark). To stop the reaction, 0.5 M sulphuric acid was added. The absorbance was measured at 405 nm using the FLUOstar Omega microplate reader (BMG Labtech, Ortenberg, Germany).

### 2.15 Colitis mouse model experiments

#### 2.15.1 DSS‐induced Colitis

Colitis model experiments were carried out following the approved guidelines of the James Cook University Animal Ethics Committee (Approval number: A2795). Groups of ten 7‐8‐week‐old male BALB/c mice (Animal Resources Centre, Australia) were treated intra‐peritoneal (i.p.) with either *A. suum* EVs (20 µg/mouse of EVs; ∼4×10^10^ particles in 230 µL PBS) or PBS vehicle only on days 0, 2, and 5 of the study. All treatment groups received DSS (MP Biomedicals, Santa Ana, CA, USA) in their drinking water (2.5% w/v solution) from day 0 until termination. Five naïve mice were used as disease‐negative controls (did not receive DSS but were injected with the same volume of PBS). To normalise the intestinal microbiota among the mice groups, the bedding from all cages was mixed and evenly distributed among the cages twice (two days apart), one week before the commencement of the experiment. Mice were weighed on days 0, 2, 5, 6, 7, and 8, and clinical scores, including assessments of piloerection, faeces consistency, and rectal thickening/injury, were blindly scored from 0 to 2, where 0 indicates normal, 1 indicates mild disease, and 2 signifies severe disease.

After euthanasia on day 8, colons were excised, and 1 cm of the distal colon was individually collected and preserved in RNA*later*™ Stabilization Solution (Invitrogen, Thermo Fisher, #AM7021). The colon samples were then drained and weighed. Five hundred (500) µL of chilled PBS was added to samples and homogenised using a TissueLyser II (QIAGEN). The level of inflammatory cytokines in the cell‐free homogenate was measured using LEGENDplex™ Mouse Inflammation Panel (BioLegend, #740446) following the manufacturer's protocol.

#### 2.15.2 TNBS‐induced colitis

Groups of ten 6‐week‐old female BALB/c mice (Australian BioResources, Moss Vale, Australia) were treated i.p. with either *A. suum* EVs (20 µg/mouse of EVs; ∼4×10^10^ particles in 230 µL PBS) or PBS vehicle only, on days ‐1 and 1. On day 0, mice in the treatment group were generally anaesthetised with 200 µL IP injection of 50 mg/kg of Ketamine (Ceva, France) and 5 mg/kg Xylazine (Troy, Australia) mix. Following anaesthesia, mice were intrarectally administered 2.5 mg of TNBS/mouse, dissolved in 50% EtOH. Three naïve mice were used as disease‐negative controls. Mouse weight was recorded daily, and clinical scores were recorded as per the protocol for DSS colitis.

### 2.16 Statistical analysis

Statistical analysis and graphs were created using GraphPad Prism v. 9.3.1 (GraphPad Software, San Diego, CA, USA). The normality of all datasets was evaluated by QQ‐plots. Groups were compared by either one‐way or two‐way ANOVA followed by the Tukey multiple comparison test. Statistical analysis of results obtained by the two T cells activity assays was conducted on data normalised to the fitting control (untreated cells) due to large variations among donors. The graphical presentation remains the raw data. *p*‐values < 0.05 were considered statistically significant. Data are expressed as means ± SD.

## 3 RESULTS

### 3.1 EV isolation and characterisation

**FIGURE 1:**
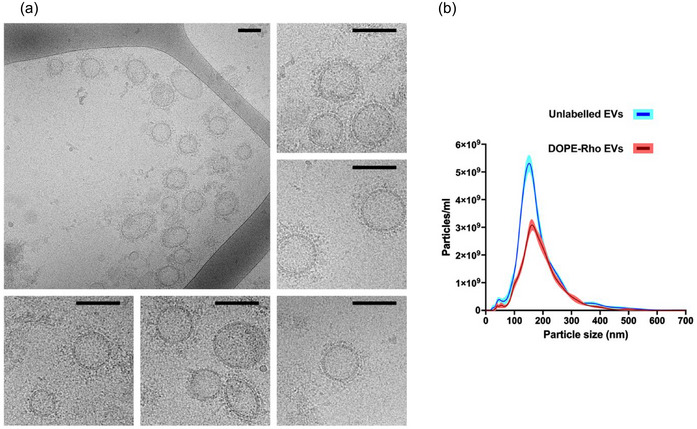
Characterization of *Ascaris suum* EVs. **(a)**. CryoTEM images of purified *Ascaris suum* EVs (40,000× magnification) showing EVs of various sizes with the presence of a visible corona. All scale bars are 100 nm. **(b)**. Size distribution profiles of unlabelled EVs and DOPE‐Rho‐labelled *A. suum* EVs acquired with NTA. The graph displays the average concentration (solid lines) and size distribution of particles (Blue: unlabelled, and red: DOPE‐Rho labelled). The standard error of the mean is expressed as shaded areas around the curves. EV, extracellular vesicles; CryoTEM, cryogenic transmission electron microscopy; NTA, nanoparticle tracking analysis.

Isolation and characterisation of EVs from *A. suum* have been validated and described in detail in our previous publication (Borup et al., 2022). Therefore, only imaging, size distribution, and concentration data are included here. Cryo‐TEM was conducted to visualise EVs and confirm the presence of lipid bilayer enclosed vesicles in EV‐enriched fractions. EVs with ∼20‐200 nm diameters and ∼30 nm peripheral filament‐like structures (i.e., corona) were observable in the Cryo‐TEM images (Figure 1a). NTA technology was used to determine EV size distribution and concentration, measuring unlabelled and DOPE‐Rho‐labelled EVs. This in‐vivo labelling technique reduces the risk of stain artefacts and non‐specific labelling and does not affect the physical properties of EVs, as demonstrated in *Anisakis* spp. (Boysen et al., 2020). The size distribution profile of both labelled and unlabelled EVs was similar, with a peak at ∼150 nm (Figure 1b). The particle concentration for unlabelled EVs was 5.86×10^11^ ± 1.36×10^10^ particles/mL, while the DOPE‐Rho EVs concentration was 3.49×10^11^ ± 3.61×10^9^ particles/mL. The EV‐depleted ESP contained 636.1 µg/mL protein, while the purified EV sample measured 342.0 µg/mL protein. The protein concentration was used to determine the corresponding amount of EV‐depleted ESP sample to the particles/cell dose of EVs for cell stimulations. The range of EV stimulations expressed in protein concentration was approximately 3–260 µg/mL (1000–100,000 particles/cell).

### 3.2 *A. suum* EV are internalised primarily by CD14^+^ monocytes

**FIGURE 2:**
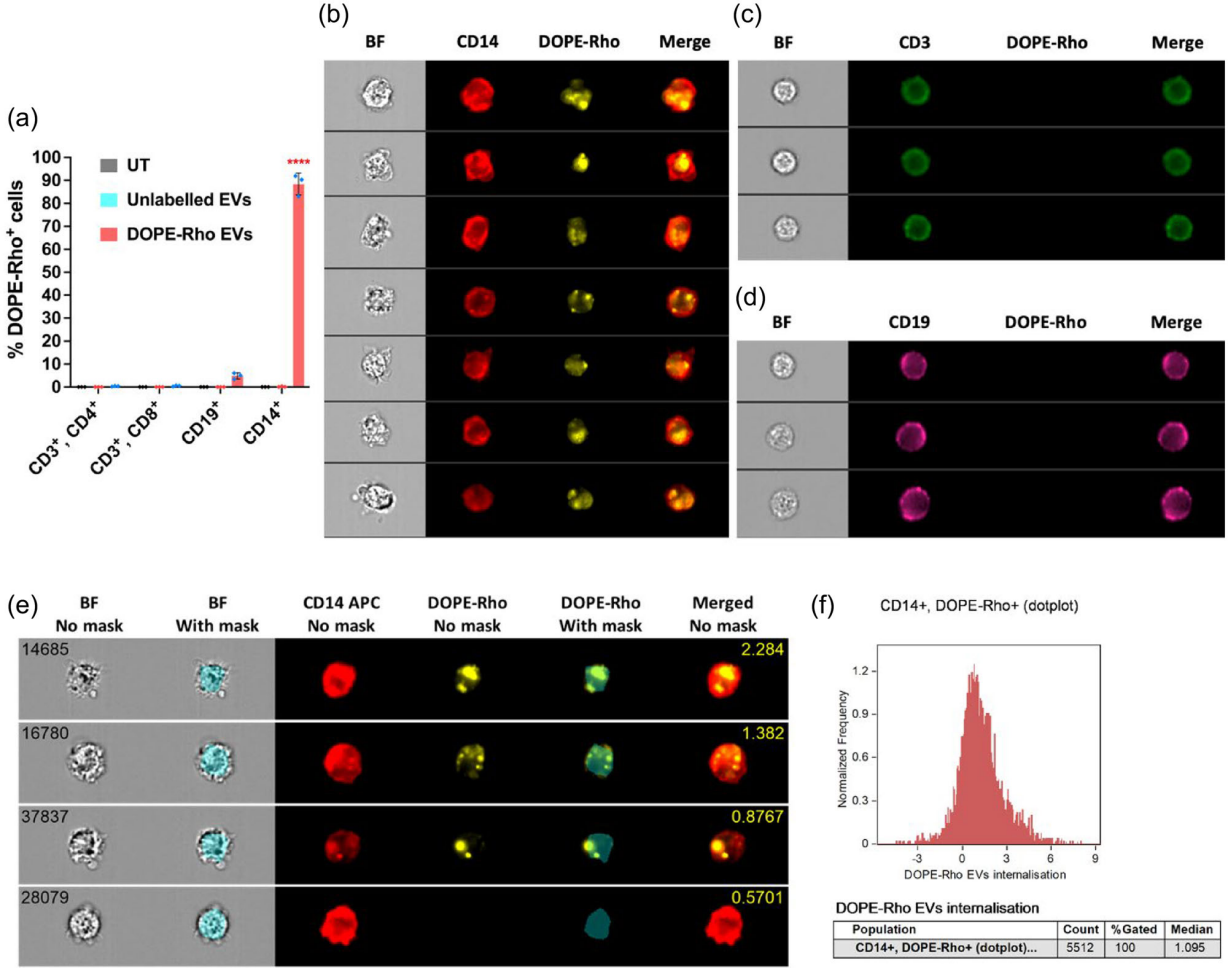
*Ascaris suum* EVs are taken up and internalised by monocytes. (**a)**. Human PBMCs were stimulated with DOPE‐Rho labelled EVs (30,000 particles/cell) and analysed by flow cytometry. UT cells and cells stimulated with unlabelled EVs were included as negative controls. The bar chart shows the percentage of EV uptake by cell type. The groups were compared using one‐way ANOVA followed by a Tukey test. ****p < 0.0001. Error bars: Mean ± SD. *n* = 3 donors. (**b, c and d)**. PBMCs incubated with DOPE‐Rho labelled EVs (30,000 particles/cell) were analysed by imaging flow cytometry. Cell surface markers for T cells (CD3^+^), B‐cells (CD19^+^) and monocytes (CD14^+^) were detected to analyse EV uptake (DOPE‐Rho^+^) in PBMCs. Monocytes (CD14^+^ cells) were significantly more positive for DOPE‐Rho compared to the other cell types and to the UT and unlabelled EVs. BF = Brightfield. (**e)**. Representative images of high and low internalisation scores. Images of one cell are shown horizontally in the brightfield channel without and with the AdaptiveErode (M01, Ch01, 75), CD14 APC, DOPE‐Rho channel without and or with the mask displayed and an image with CD14APC and DOPE‐Rho merged. The internalisation score is shown as yellow numbers on the merged images. (**f)**. Accumulated internalisation score for all CD14^+^, DOPE‐Rho^+^ cells plotted against the normalised frequency. The internalisation of DOPE‐Rho EVs in CD14^+^ monocytes was determined using the mask shown in e. Created with IDEAS Software. EV, extracellular vesicles; PBMCs, peripheral blood mononuclear cells; UT, untreated.

We have previously shown that *A. suum* EVs downregulate LPS‐induced inflammation in human PBMCs (Borup et al., 2022). To further explore this effect, we investigated which cell type/s in PBMCs internalise *A. suum* EVs. Human PBMCs were stimulated with labelled EVs (DOPE‐Rho EVs) or unlabelled EVs and analysed with specific makers for T cells (CD3^+^, CD4^+^, CD8^+^), B cells (CD19^+^) and monocytes (CD14^+^) using flow cytometry. DOPE‐Rho EVs were primarily taken up by monocytes, whilst minimal uptake was observed for B cells and T cells. Of the B‐cells, 0.2% were DOPE‐Rho^+^, however, these were revealed to be artefacts by visualisation (data not shown). The percentage of DOPE‐Rho^+^ monocytes was 80–90% of all CD14^+^ cells and significantly higher compared to the other cell types (Figure 2a).

Imaging flow cytometry using the ImageStream®X MKII was performed to validate whether the EVs were internalised or adhered to the cell membrane of the monocytes. Confirming the findings by flow cytometry, the lymphocyte populations (CD3^+^ and CD19^+^ cells) were DOPE‐Rho^−^ (Figure 2a, 2c and 2d). CD14^+^ monocytes were DOPE‐Rho^+^, supporting the previous findings showing uptake of EVs specifically by monocytes (Figure 2a and 2b). To understand if the EVs were binding to or internalised by the cells, we created the mask *AdaptiveErode (M01, Ch01, 75)* (Figure 2f) to define the inside of the cells. We combined this mask with the internalisation feature, defined as the ratio between the DOPE‐Rho intensity inside the cell and the intensity of the entire cell. Cells with internalised EVs will have a positive score, while cells with little or no internalisation typically have a negative score. For CD14^+^, and DOPE‐Rho^+^ cells, the median internalisation score was 1.10 (Figure 2e). We, therefore, conclude that EVs were internalised by monocytes.

### 3.3 *A. suum* EV stimulation alters the expression of PD‐L1 and CCR2 and the composition of monocyte subpopulations

**FIGURE 3:**
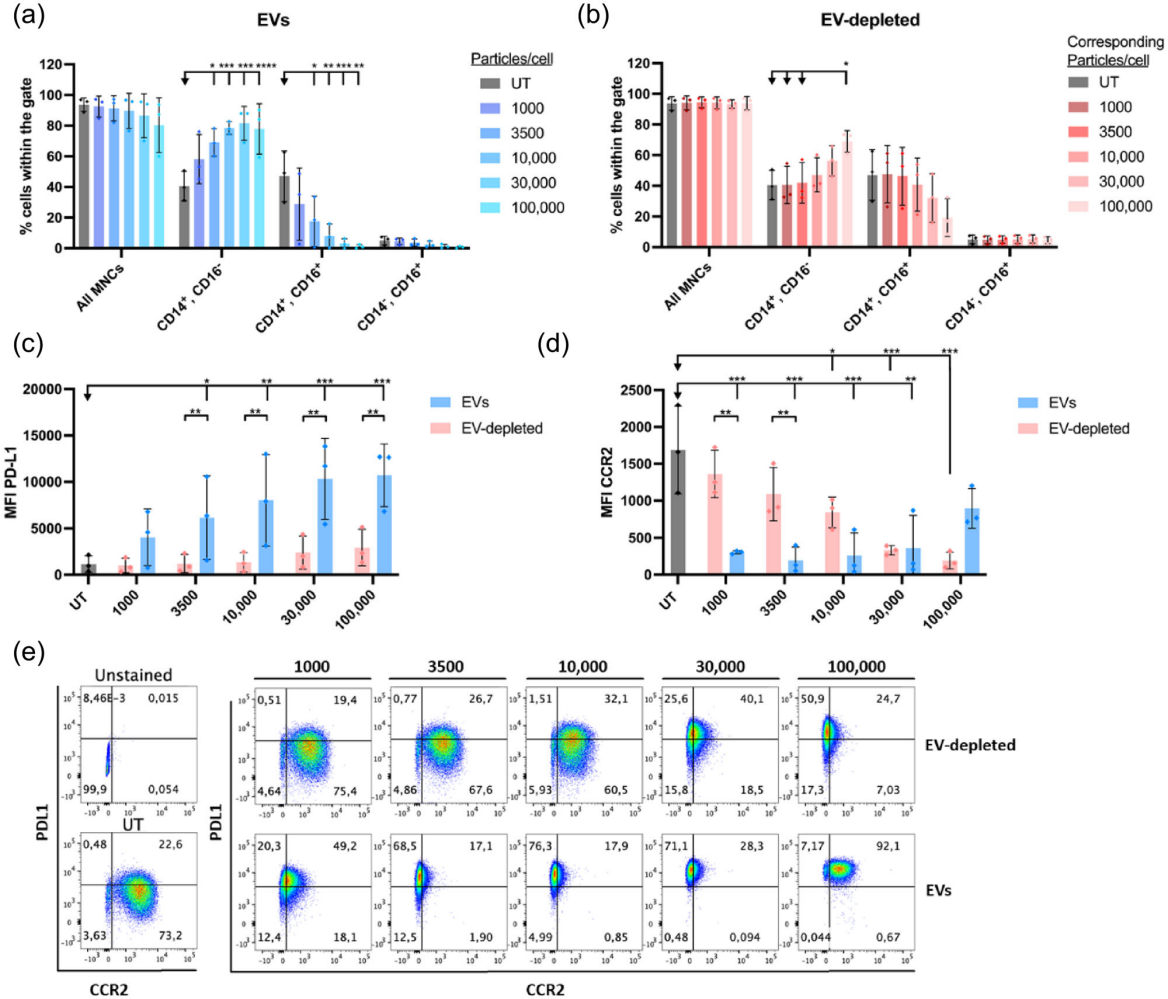
*Ascaris suum* EVs reduced the percentage of CD14+, CD16+ cells, increased CD14+, CD16− cells, and modulated the expression of PD‐L1 and CCR2**. (a) and (b)**. Human PBMCs stimulated with EVs (A) or EV‐depleted ESP (B), and the subpopulations of monocytes (classical: CD14^+^, CD16^−^; intermediate: CD14^+^, CD16^+^; non‐classical monocytes: CD14^−^, CD16^+^) were analysed by flow cytometry. A group containing all MNCs represents the combined subpopulations. Exposure of monocytes to increasing numbers of particles/cell was undertaken; the EV‐depleted ESP was normalised by protein concentration to the equivalent number of EVs for comparative purposes. The (UT) cells were incubated with culture media alone. The groups were compared using two‐way ANOVA followed by a Tukey test. *p < 0.05, **p < 0.01, ***p < 0.001. Error bars: Mean ± SD. *n* = 3 donors. **(c) and (d)**. The MFI of PD‐L1 (c) and CCR2 (d) expression on all MNCs was analysed by flow cytometry on human PBMCs stimulated with either EVs or EV‐depleted ESP. The groups were compared using two‐way ANOVA followed by a Tukey test. *p < 0.05, **p < 0.01, ***p < 0.001, ****p < 0.0001. Error bars: Mean ± SD. *n* = 3 donors. **(e)**. Dotplot of PD‐L1 and CCR2 co‐expression in one representative donor untreated, stimulated with EVs or EV‐depleted ESP. ESP, excretory‐secretory products; EV, extracellular vesicles; MFI, median fluorescence intensity; MNCs, mononuclear cells; PBMC, peripheral blood mononuclear cells; PD‐L1, programmed death‐ligand 1.

Based on the efficient internalisation of EVs by monocytes, we investigated whether EVs mediate phenotypic changes in monocytes. This was done by analysing the expression of specific surface markers of known monocyte phenotype subsets implicated in inflammatory responses. Human PBMCs were stimulated with EVs (1,000‐100,000 particles/cell) or EV‐depleted ESP (added at protein concentrations normalised to EV protein levels) for 16–18 h and analysed by flow cytometry using a panel of monocyte characterisation and activation markers. Monocytes were subdivided into the classical (CD14^+^, CD16^−^), intermediate (CD14^+^, CD16^+^) and non‐classical monocytes (CD14^−^, CD16^+^). The percentage of intermediate (CD14^+^, CD16^+^) monocytes significantly declined in a concentration‐dependent manner with increasing number of EVs (Figure 3a, Figure S2A), concomitant with a concentration‐dependent increase in the percentage of classical monocytes (CD14^+^, CD16^−^). Importantly, these effects were not replicated in monocytes co‐cultured with EV‐depleted ESP, except in the highest concentration (Figure 3b, Figure S2B).

The analysis of monocyte activation markers was conducted on the total monocyte population (defined as “All MNCs”, gating in Figure S3B). We observed that PD‐L1, a well‐described checkpoint of the immune system, showed a dose‐dependent increase in expression compared to both the negative control and the EV‐depleted control (Figure 3c). All stimulations with EVs, with the exception of 1,000 particles/cell, showed significantly higher expression of PD‐L1 compared to the untreated control cells. The three highest concentrations of EVs (10,000, 30,000, and 100,000 particles/cell) showed significantly higher PD‐L1 expression compared to monocytes treated with the equivalent concentrations of EV‐depleted ESP, confirming the role of EVs in driving PD‐L1 expression.

Conversely, monocyte expression of CCR2 was significantly reduced upon EV stimulation (Figure 3d) at the lowest number of EVs (1000 particles/cell), and this effect was maintained at 3500, 10,000, and 30,000 particles/cell, whilst the highest concentration (100,000 particles/cell) did not induce a significant decrease in CCR2 expression. EV‐depleted ESP resulted in a concentration‐dependent reduction in monocyte CCR2 expression, but this effect was limited to higher protein concentrations that corresponded to 10,000, 30,000, and 100,000 particles/cell. At lower particle concentrations (1,000 and 3,500 particles/cell), a significant difference in monocyte CCR2 expression was observed between EV and EV‐depleted ESP samples, suggesting that whilst EV and EV‐depleted fractions are capable of inducing reductions of CCR2, EVs are more efficient. Interestingly, most concentrations of EVs resulted in a CCR2^−/low^/PDL1^+/High^ monocyte population, except the highest concentration of EVs (Figure 3e, Figure S10).

Aside from PD‐L1 and CCR2, the expression of HLA‐DR and CD86 in the panel was unaffected between EV depletion and EVs (Figure S3A).

### 3.4 PNGase treatment of *A. suum* EV does not alter their functional effects on monocytes

**FIGURE 4:**
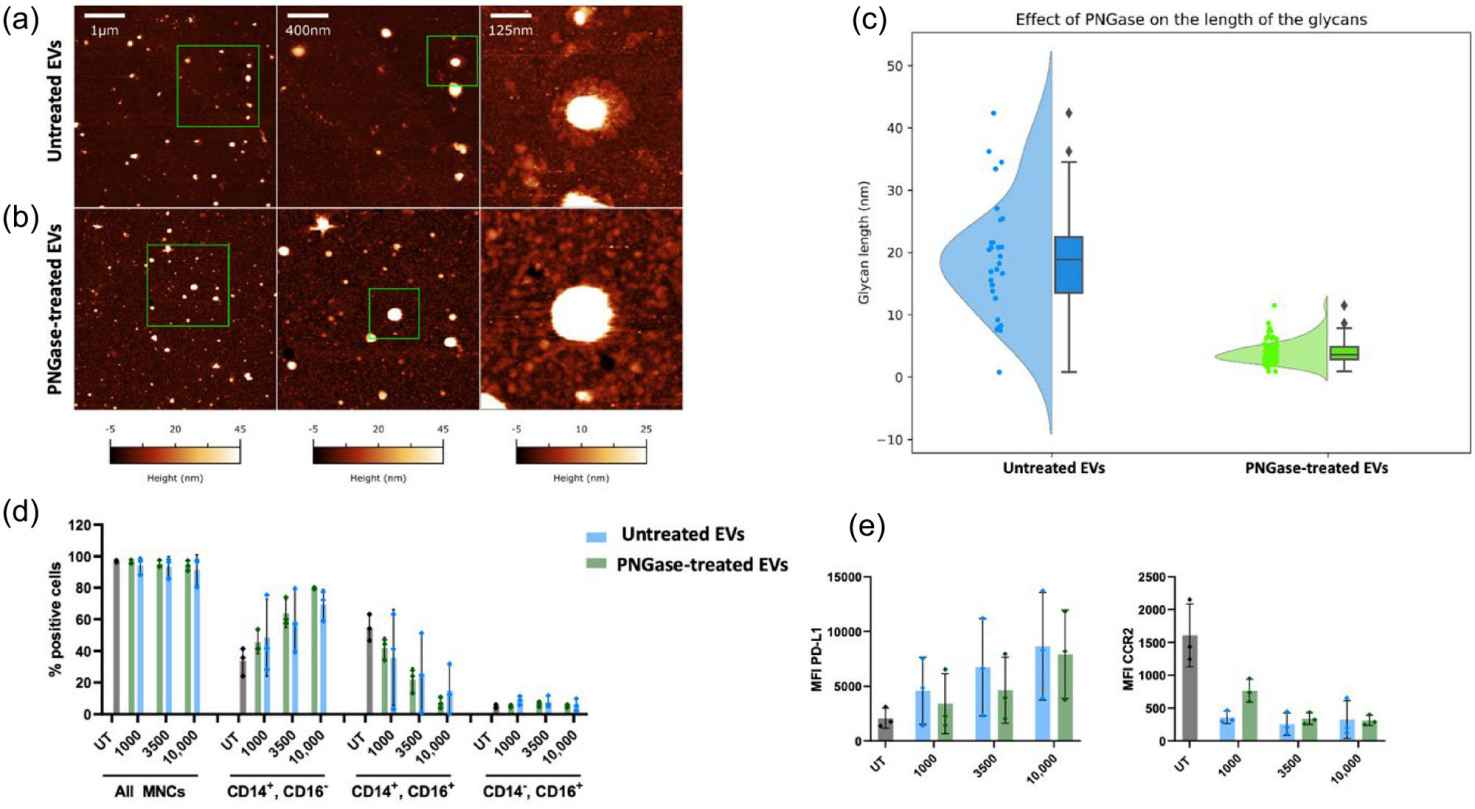
Removal of N‐linked glycans on the surface of *Ascaris suum* EVs did not alter their effect on human PBMCs. **(a) and (b)**. AFM micrographs of untreated (a) and 400 U/mL PNGase F treated (b) *Ascaris suum* EVs. Untreated EVs appear to be decorated by a diffuse corona of filaments, which mostly disappears after PNGase treatment. the process does not compromise the structural integrity of EVs. Further details and quantitative measurements can be found in Figure S‐10. **(c)**. Distribution of the glycoprotein corona thickness as measured via AFM on individual untreated (blue) and PNGase‐treated *A. suum* EVs (green). **(d) and (e)**. The effect of PNGase and untreated EVs on the monocyte subgroups (Classical: CD14^+^, CD16^−^, Intermediate: CD14^+^, CD16^+^, Non‐classical: CD14^−^, CD16^+^) or the MFI of PD‐L1 (C) or CCR2 (D) were similar to untreated EVs. EVs were given in particles/cell doses (1000, 3500, or 10,000 particles/cell). The UT was culture media alone. Error bars: Mean ± SD. *n* = 3 donors. AFM, atomic force microscopy; EV, extracellular vesicles; MFI, median fluorescence intensity; UT, untreated cells.

In accordance with CryoTEM measurements (Figure 1), AFM micrographs of individual EVs from *A. suum* confirmed the presence of a ∼20 nm‐thick corona composed of filament‐like structures extending around the EV membrane (Figure 4a). These structures were suspected to be glycoproteins, as previously described for *Schistosoma mansoni* EVs (Kuipers et al., 2020). To confirm this hypothesis, *A. suum* EVs were treated with peptide:N‐glycosidase F (PNGase F), which enzymatically removes N‐linked glycans. PNGase F treatment successfully removed most of the filament‐like structures whilst preserving the structural integrity of the bilayer (Figure 4b, Figure S11), thereby excluding any major contribution by the corona to the structural stability of the EVs. After PNGase F treatment, the average thickness of the glycoprotein corona decreased from 19 ± 10 nm to 4 ± 2 nm (Figure 4c). Helminth glycans and helminth EV glycans have been postulated to play a role in host‐immune modulation (Tawill et al., 2004; Tundup et al., 2012; Whitehead et al., 2020); therefore, we investigated whether the glycoprotein corona had a role in the observed functional effects, assessed by flow cytometry of PBMCs stimulated with EVs and PNGase treated (’shaved’) EVs for comparison using the same monocyte antibody panel as described above (Figure 4d and 4e). No difference was observed between EVs and PNGase‐treated EVs based on monocyte subgroups or expression of PD‐L1 and CCR2. Therefore, the role of N‐linked glycan filament‐like structures was determined to be dispensable for the observed effects of EVs on monocyte phenotype.

### 3.5 Proteolytic cleavage partially mediated the downregulation of CD16 expression after EV stimulation

**FIGURE 5:**
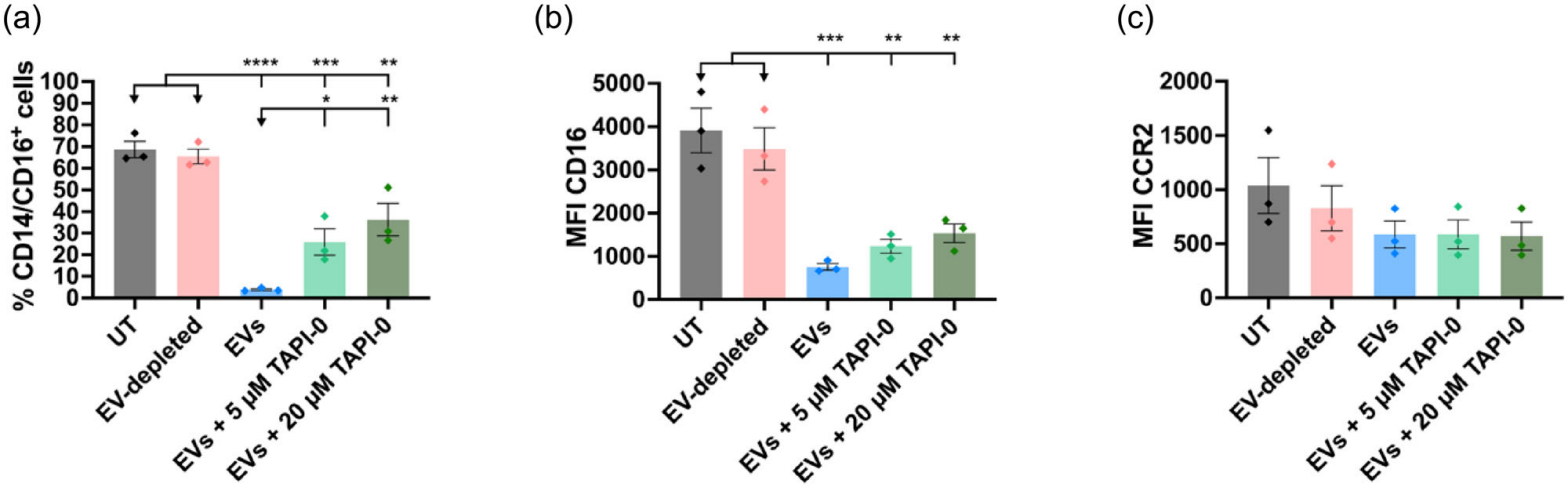
Proteolytic inhibition partially recovered EV‐dependent loss of CD16 expression. (a), (b) and (c). Human PBMCs were stimulated with Ascaris suum EVs (30,000 per cell) and two different concentrations of the metalloprotease inhibitor TAPI‐0 (5 µM and 20 µM) and analysed using flow cytometry. UT cells and EV‐depleted ESP‐treated cells were included as controls. The percentage of CD14+/CD16+ cells (a) or the MFI of CD16 (b) after stimulation with the different treatments was assessed. The MFI of CCR2 expression after stimulation with the different treatments (c). The groups were compared using one‐way ANOVA followed by a Tukey test. *p < 0.05, **p < 0.01, ***p < 0.001, ****p < 0.0001. Error bars: Mean ± SD. *n* = 3 donors. ESP, excretory‐secretory products; EV, extracellular vesicles; MFI, median fluorescence intensity; PBMCs, peripheral blood mononuclear cells; UT untreated.

Previously, it was shown that metalloproteases had an active role in the loss of intermediate monocytes after LPS stimulation (CD14^+^, CD16^+^) (Waller et al., 2019). Also, the metalloprotease ADAM17 has been found in human exosomes (Cardeñes et al., 2021). Hence, following our findings regarding the decline of CD16^+^ monocytes upon exposure to *A. suum* EVs, we investigated whether this was mediated by proteolytic activity caused by the EVs. Therefore, PBMCs were incubated with EVs for a shorter period of 30 min in the presence or absence of the broad‐spectrum metalloproteinase inhibitor TAPI‐0. The addition of TAPI‐0 significantly recovered the percentage of CD14^+^ and CD16^+^ cells compared to cells stimulated with EVs in the absence of the protease inhibitor (Figure 5a). The expression of CD16 showed no significant difference upon the addition of either 5 µM (*p* = 0.75) or 20 µM TAPI‐0 (*p* = 0.37) (Figure 5b). Both the percentage of CD14^+^ and CD16^+^ cells and expression of CD16 remained significantly higher for the controls untreated (UT) and EV‐depleted, compared to the three EV‐stimulated samples. The role of metalloproteinases was also investigated for the downregulation of CCR2 expression, but TAPI‐0 treatment showed no effect (Figure 5c).

### 3.6 *A. suum* EV affected T cell activity in a monocyte‐dependent manner

**FIGURE 6:**
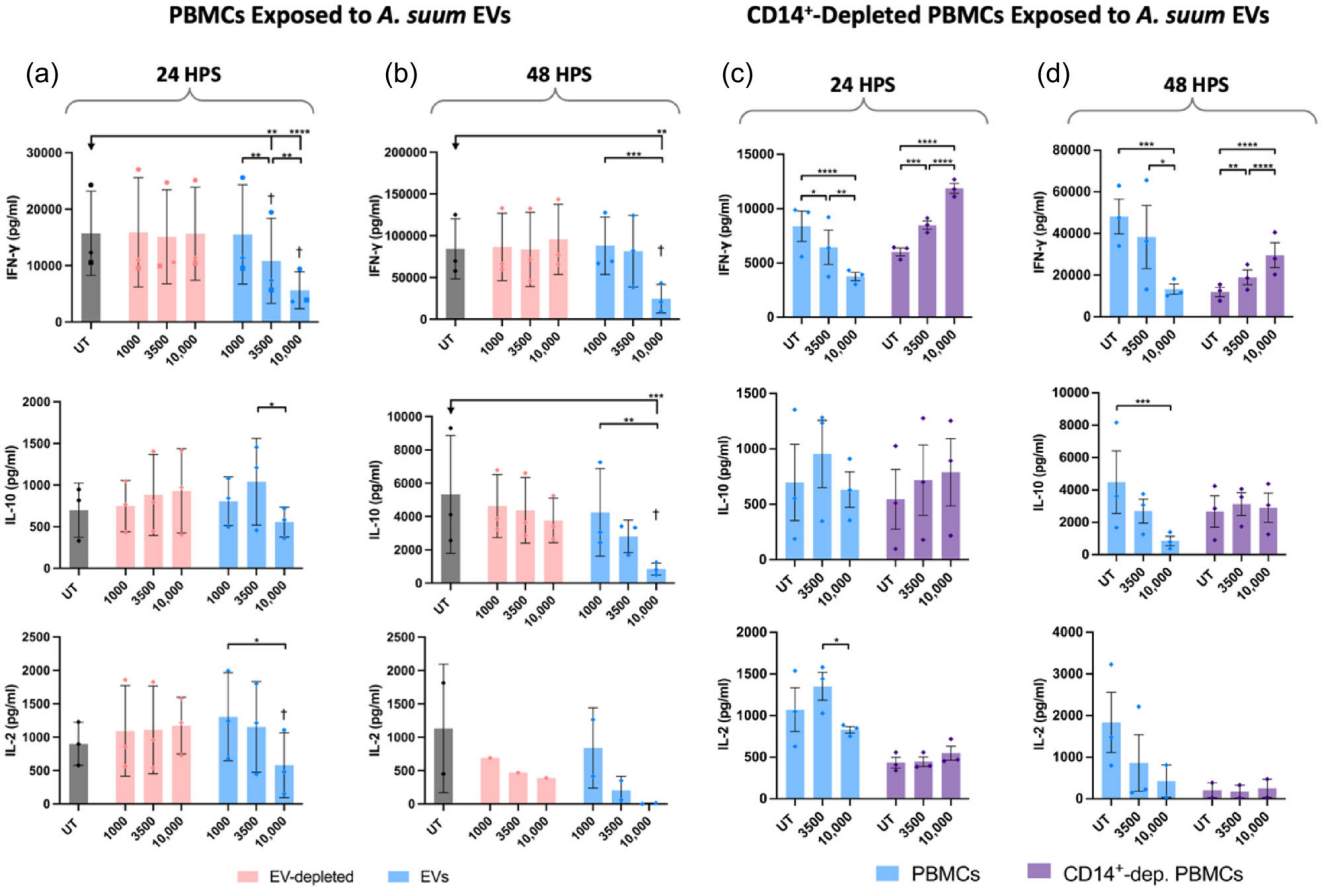
*Ascaris suum* EVs reduce T cell activity in a monocyte‐dependent manner. (a) and (b). Human PBMCs were treated with anti‐CD3/anti‐CD28 after 16 h of incubation with EVs (1,000, 3,500, and 10,000 particles/cell) or the equivalent protein concentration of EV‐depleted ESP. Cytokines were analysed either 24 h post‐stimulation (HPS) (a) or 48 HPS (b). UT cells were included as a control. Before the statistical analysis, the data were normalised to the UT group due to the large donor‐to‐donor variation. Afterwards, the groups were compared using Two‐way ANOVA followed by a Tukey test. *p < 0.05, **p < 0.01, ***p < 0.001, ****p < 0.0001, † = Significant different from the corresponding EV‐depleted dose. Error bars: Mean ± SD. *n* = 3 donors. (c) and (d). PBMCs were depleted of CD14^+^ cells before being stimulated with EVs (3500 and 10,000 particles/cell) and subsequently activated with anti‐CD3/anti‐CD28. Cytokines were analysed either 24 HPS (c) or 48 HPS (d). UT cells and non‐depleted PBMCs from the same donor were included as a control. Before the statistical analysis, the data were normalised to the UT group due to the large donor‐to‐donor variation. Afterwards, the groups were compared using two‐way ANOVA followed by a Tukey test. *p < 0.05, **p < 0.01, ***p < 0.001, ****p < 0.0001. Error bars: Mean ± SD. *n* = 3 donors. ESP, excretory‐secretory products; EV, extracellular vesicles; MFI, median fluorescence intensity; PBMCs, peripheral blood mononuclear cells; UT untreated.

As the phenotypic changes in monocytes induced by EV stimulation, such as increased surface expression of PD‐L1, may affect the activity of T cells in a mechanism similar to that commonly observed by monocyte/macrophage PD‐L1 in cancer (Iwai et al., 2002; Yasuoka et al., 2020), we stimulated PBMCs with *A. suum* EVs in the presence of anti‐CD3/CD28 antibodies to activate T cells or isotype‐matched control antibodies. Supernatants were analysed after 24 and 48 h post‐stimulation (HPS) for common T cell cytokines (IFNγ, IL‐2, IL‐10). At 24 HPS, the release of all three cytokines was significantly reduced in a concentration‐dependent manner by EV stimulation (Figure 6a). The results followed the same pattern at 48 HPS cells with the exception of IL‐2, as some concentrations were below the detection limit despite the use of the same donors (Figure 6b). EV‐depleted ESP did not elicit the same effect, regardless of incubation time. The decrease in cytokine levels was not due to cell death, as viability and cell count were stable for all stimulated cells compared to UT control cells (data not shown). Isotype controls were also included to evaluate non‐specific Fc‐binding and activation of immune cells, with no effect on cytokine production (data not shown).

Moreover, the impact of monocytes on T cell activation and EV stimulation was analysed by depleting CD14^+^ cells from PBMCs prior to stimulation (Figure 6c and d). The lowest EV concentration (1,000 particles/cell) was excluded as the previous experiment showed little to no effect on PBMCs at this dose (Figure 6a and b). IFN‐γ levels significantly increased for EV‐stimulated CD14^+^‐depleted PBMCs for both time points, whilst monocyte‐replete PBMCs showed a significant reduction of IFN‐γ (Figure 6c and d). Similarly, after 48 HPS, IL‐10 levels from the complete PBMCs were significantly reduced by the highest EV concentration (10,000 particles/cell) to less than 22% of the UT control (Figure 6b). The same effect was not observed for CD14‐depleted PBMCs. The levels of IL‐2 after 24 HPS showed a significant difference between 3,500 and 10,000 particles/cell dose (Figure 6c). The analysis of IL‐2 levels after 48 HPS was inconclusive as the CD14^+^‐depleted PBMCs and one donor for the 10,000 particle/cell stimulation for complete PBMCs presented with levels lower than the limit of detection (Figure 6d). Combined, this shows that EV‐mediated attenuation of T cell activation is dependent on monocytes.

### 3.7 *A. suum* EV mitigate colitis symptoms in the DSS mouse model but not in the TNBS model

To investigate the immunomodulatory/anti‐inflammatory effects of *A. suum* EVs in‐vivo, we treated mice with EVs and assessed their prophylactic properties in two distinct models of inducible colitis. TNBS‐induced and DSS‐induced colitis are widely utilised models of T cell‐dependent and T cell‐independent colitis, respectively, where chemical damage to the intestine triggers immunological features that are representative of those seen in human IBDs (Kiesler et al., 2015).

**FIGURE 7:**
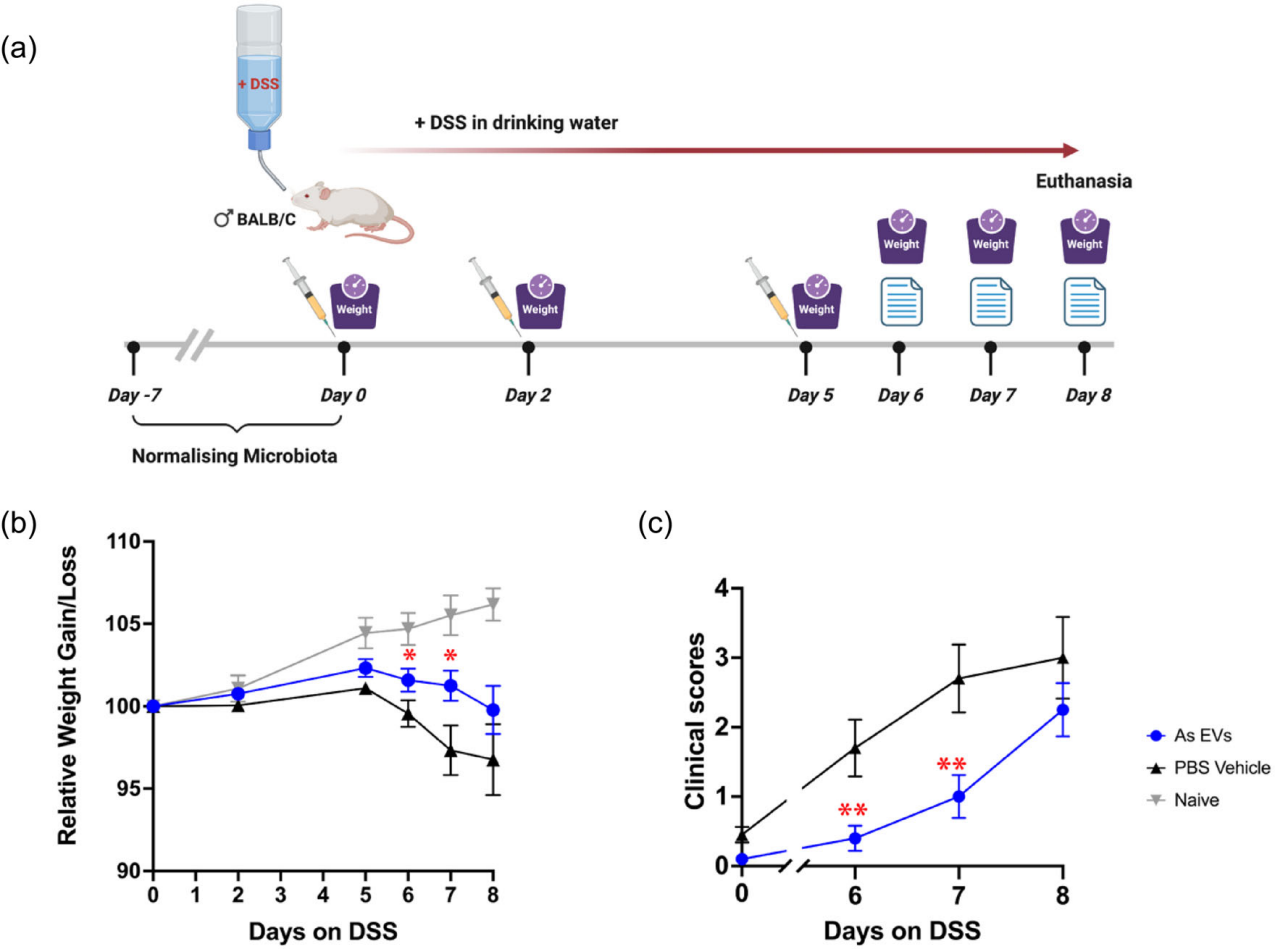
Mitigating effects of *Ascaris suum* EVs on DSS colitis. (a). Mice received three i.p. injections on days 0, 2, and 5 (20 µg per injection for the *Ascaris suum* EV treatment group). Starting from day 0, mice were subjected to 2. 5% (w/v) DSS in their drinking water until euthanised on day 8, with continuous recording of body weight and clinical scores. (**b)**. Relative weight gain/loss for mice during the study. Statistical comparisons between the treatment and PBS vehicle groups were conducted using the Mann‐Whitney test; * *p* < 0.1; (**c)**. Cumulative clinical scores encompassing piloerection, faeces consistency, and rectal thickening/injury on days 0, 6, 7 and 8 of the study. Statistical comparisons between the treatment and PBS vehicle groups were conducted using the Kruskal‐Wallis test; * *p* < 0.05; ** *p* < 0.01. EV, extracellular vesicles i.p, intraperitoneali.

In the DSS colitis study, mice treated with PBS vehicle started losing weight from day 5 post‐DSS administration, whereas mice treated with *A. suum* EVs exhibited significant improvement in maintaining their body weight. Mice in the naïve group (regular drinking water without DSS) maintained their gradual weight gain throughout the study (Figure 7a and b). Clinical scores data aligned with the body weight data, where mice treated with *A. suum* EVs exhibited lower scores (mitigated symptoms) compared to the PBS vehicle control group on days 6 and 7 (Figure 7c).

DSS colitis resulted in elevated levels IL‐6, IL‐1a, TNF‐ɑ, and MCP‐1 in the colons of PBS control‐treated mice compared to naïve mice (Figure S4). Mice treated with *A. suum* EVs displayed a non‐significant trend towards reduced IL‐6 levels in colon tissue compared to the PBS vehicle group, while levels of other cytokines remained unaffected.

In the TNBS‐induced colitis study, the PBS vehicle control group and the *A. suum* EV treated groups did not show any significant difference in their body weight loss, both losing up to 15% of their body weight over a three‐day period (Figure S5, A and B). Clinical scores also indicated no difference between the two groups (Figure S5, C).

## 4 DISCUSSION

In summary, this study has demonstrated that the EVs released by *A. suum* are efficiently internalised by monocytes and drive phenotypic changes, including up‐regulation of PD‐L1. In a monocyte‐dependent manner, the EVs attenuate T cell activation in PBMCs and protect against DSS‐induced colitis in mice.

Uptake of mammalian EVs by monocytes or macrophages has already been shown, but in contrast to our data, human EVs showed significant uptake in B cells as well as monocytes (Eitan et al., 2017; Rutman et al., 2018). Whilst other studies have demonstrated the uptake of helminth EVs by monocyte/macrophage cell lines or bone marrow‐derived mouse macrophages, this is the first report of EV internalisation by monocytes in a mixed population of primary human immune cells (Ben Ami Pilo et al., 2022; Boysen et al., 2020; Coakley et al., 2017; Kifle et al., 2020; Liu et al., 2019).

Given that the monocytes were the sole target of *A. suum* EVs in human PBMCs, we analysed the expression profile of EV‐stimulated monocytes. Surprisingly, EVs induced a dose‐dependent downregulation of CD16 on the surface of monocytes in a mechanism partially dependent on metalloprotease activity. This finding is consistent with a previous observation that the loss of intermediate monocytes (CD14^+^, CD16^+^) after LPS stimulation was prevented by an ADAM17‐inhibitor (Waller et al., 2019). Although metalloproteinases, including ADAM17, have been shown to be present in human EVs (Cardeñes et al., 2021) our data cannot determine if *A. suum* EVs are carriers of a similar metalloproteinase or activate a host enzyme in monocytes. Regardless, this activity resulted in the loss of intermediate monocytes (CD14^+^, CD16^+^) concomitant with an increase in classical monocytes (CD14^+^, CD16^−^). *A. suum* EVs also resulted in downregulation of CCR2 whilst upregulating the immune checkpoint PD‐L1. Of note, we found that a dose of *A. suum* EVs as low as 1,000 particles/cell significantly downregulated CCR2 in a rapid manner. Classical monocytes (CD14^+^, CD16^−^) are characterised by high CCR2 levels whereas high PD‐L1 expression is associated with non‐classical (CD14^−,^ CD16^+^) monocytes (Lehman et al., 2023). Therefore, we propose that *A. suum* EVs induce a novel monocyte phenotype characterised as CD14^+^/CD16^−^/CCR2^−/low^/PDL1^+/High^. We also detected lesser changes at the highest amounts of EV‐depleted ESP, which may correspond to EV‐vesicles not being completely depleted by size exclusion chromatography (Borup et al., 2022).

Changes in monocyte phenotype are critical for the host since, for example, the expansion of CD16^+^ monocytes is associated with several inflammatory conditions (Wong et al., 2012). Increasing amounts of CD16^+^ monocytes were observed in IBD and neonatal enterocolitis, confirming a pro‐inflammatory role for these cells in the gastrointestinal tract (Koch et al., 2010; Olaloye et al., 2021). CD16^+^ monocytes are also involved in the proliferation and stimulation of T cells, especially CD4^+^ T cells and migrate more often to tissue with inflammation (Ożańska et al., 2020). Similarly, CCR2 has a crucial role in the pro‐inflammatory effects of monocytes as the key cell surface receptor for the recruitment and transmigration of monocytes to inflamed or infected tissue, as well as the replenishment of intestinal macrophages during homeostasis (Desalegn & Pabst, 2019). As such, CCR2 is a key component for the development of IBD, and the absence of the receptor or its cognate ligand CCL2, leads to reduced disease severity in mice models (Bain & Mowat, 2014; El Sayed et al., 2022; Pei et al., 2017).

Taken together, this indicates that *A. suum* EVs target both monocyte inflammatory state and chemotaxis to sites of inflammation, and as such, helminth EVs could serve as a novel therapeutic modality for inflammatory diseases, which is supported by previous reports (Long et al., 2021; Roig et al., 2018; Wangchuk et al., 2019).

In addition to these direct effects on the monocyte inflammatory state, we also show EV‐mediated up‐regulation of an immune checkpoint that can influence the immune activation of other immune cell populations. The PD‐L1/PD‐1 axis serves as an immune checkpoint leading to T cell anergy and provides co‐suppressive signals during the T cell receptor (TCR) activation (Bennett et al., 2003). The importance of this axis is apparent in cancer and has led to the development of therapies blocking programmed cell death protein 1 (PD‐1) mediated T cell anergy to restore cytotoxic T cell anti‐cancer responses (Latchman et al., 2004; Liu et al., 2021). The PD‐L1/PD‐1 axis is also considered important for the progression of multiple sclerosis (MS). A certain PD‐1 polymorphism is linked to disease progression with increased IFN‐γ T cell secretion, and PD‐L1^+^IL‐10^+^CD14^+^ cells are found to be significantly increased during disease remission (Kroner et al., 2005; Trabattoni et al., 2009). Similarly, PD‐L1 blockade during *Leishmania* spp. infection improved immune response and reduced parasite burden (Jafarzadeh et al., 2022). Upregulation of PD‐L1 by helminths has previously been shown. For example, *Brugia malayi* microfilariae lysate increased PD‐L1 expression on monocytes in vitro (O'Regan et al., 2014; Venugopal et al., 2017) and in macrophages from *S. mansoni* infected mice, antibody‐blockade of PD‐1/PD‐L1 prevented T cell anergy (Smith et al., 2004). However, to the best of our knowledge this is the first evidence of helminth EVs inducing PD‐L1 on monocytes to drive T cell anergy. As this anergy is monocyte‐dependent and the EVs are internalised in the monocytes, the effect could be caused by lack of antigen‐presentation in the monocyte‐T cell interplay. The decrease in IFN‐γ and IL‐2 levels is consistent with the general observation that helminths suppress pro‐inflammatory type 1 response in favour of a modulated type 2 response (Zakeri et al., 2018) and consistent with attenuated TNF, IL‐12, and IFN‐γ production by mitogen‐stimulated T cells isolated from *A. lumbricoides*‐infected people (Geiger et al., 2002). Furthermore, we have previously shown that *A. suum* EVs suppress LPS‐induced TNF release from PBMCs (Borup et al., 2022). The potent in vitro suppression of IFN‐γ by *A. suum* EVs suggests that they could serve as therapeutics for autoimmune diseases, as dysregulation of IFN‐γ promotes inappropriate inflammation such as that present in IBD (Langer et al., 2019).

Lastly, we assessed the immunomodulatory/anti‐inflammatory effects of *A. suum* EVs in‐vivo, using DSS‐induced and TNBS‐induced colitis mouse models that induce pathology via distinct but highly relevant cellular mechanisms, monocyte/macrophage and T cell driven, respectively. TNBS‐induced colitis mirrors human Crohn's disease, involving T cell mediated inflammation and intestinal transmural inflammation. In contrast, DSS induces colitis without T cell involvement, but cytokines from innate cells (specifically monocytes and macrophages) are sufficient for driving intestinal inflammation ^29^. We found that i.p. injection of *A. suum* EVs into mice mitigated colitis induced by DSS. This supports our in vitro studies, where *A. suum* EVs selectively target peripheral monocytes and drive a phenotype that is anti‐inflammatory, but crucially for DSS‐induced colitis, may prevent monocyte recruitment and, therefore, tissue damage mediated by pro‐inflammatory macrophages (Pei et al., 2017). Moreover, in the post‐mortem cytokine study, we observed a trend towards reduced IL‐6 levels in colon tissue compared to the PBS vehicle group. Previous studies indicated that IL‐6 inhibition could ameliorate intestinal permeability in DSS‐induced colitis through the reduced expression of the intestinal tight junction protein, claudin‐2 (Xiao et al., 2016).

Conversely, *A. suum* EVs did not attenuate TNBS‐induced colitis, and this was surprising given the potent inhibition of T cell activation mediated by *A. suum* EVs in vitro and the aforementioned role of T cells in the pathology of this model. It is possible that the same mechanisms we predict to be providing beneficial effects in DSS‐induced colitis may result in the failure of *A. suum* EVs in TNBS‐induced colitis, for example, the EV‐mediated and rapid downregulation of CCR2 likely prevents recruitment of anti‐inflammatory monocytes to the intestine that we have shown were crucial for attenuation of T cell responses. Moreover, TNBS triggers an extremely acute form of colitis involving a larger number of cells in the pathogenesis, and its effects are nearly instantaneous post‐induction. Therefore, the prophylactic effects of the *A. suum* EVs on TNBS might have required a more prolonged exposure to take place.

In conclusion, this work suggests a pivotal and targeted role for *A. suum* EVs in hijacking immune pathways that are governed by monocytes/macrophages, and we therefore present EVs as new players in T cell anergy observed in *A. lumbricoides* infected individuals. The targeting of professional phagocytes to induce both direct and indirect pathways of immune modulation presents a highly novel and efficient mechanism of EV‐mediated host‐parasite communication. Moreover, the anti‐inflammatory effects of *A. suum* EVs were translatable in‐vivo and reduced clinical disease parameters in DSS colitis, indicating the presence of molecular moieties with drug‐like properties, and highlighting the potential of helminths and their secretions for treating diseases that result from dysregulated immune system.

## AUTHOR CONTRIBUTIONS

**Anne Borup**: Conceptualization (equal); data curation (equal); formal analysis (equal); investigation (equal); methodology (equal); software (equal); validation (equal); visualization (equal); writing—original draft (equal); writing—review and editing (equal). **Mohammad Farouq Sharifpour**: Conceptualization (equal); data curation (equal); formal analysis (equal); investigation (equal); methodology (equal); software (equal); validation (equal); visualization (equal); writing—original draft (equal); writing—review and editing (equal). **Litten S. Rossen**: Conceptualization (supporting); formal analysis (supporting); investigation (supporting); methodology (supporting); software (supporting); writing—review and editing (supporting). **Bradley Whitehead**: Conceptualization (supporting); methodology (supporting); writing—review and editing (supporting). **Anders T. Boysen**: Investigation (supporting); methodology (supporting); visualization (supporting); writing—review and editing (supporting). **Rikke Olesen**: Resources (supporting); writing—review and editing (supporting). **Anja B. Bohn**: Formal analysis (supporting); methodology (supporting); writing—review and editing (supporting). **Andrea Ridolfi**: Investigation (supporting); methodology (supporting); writing—review and editing (supporting). **Marco Brucale**: Formal analysis (supporting); investigation (supporting); methodology (supporting); validation (supporting); writing—review and editing (supporting). **Francesco Valle**: Methodology (supporting); resources (supporting); writing—review and editing (supporting). **Lucia Paolini**: Investigation (supporting); methodology (supporting); writing—review and editing (supporting). **Annalisa Radeghieri**: Investigation (supporting); methodology (supporting); writing—review and editing (supporting). **Paolo Bergese**: Funding acquisition (supporting); resources (supporting); writing—review and editing (supporting). **Kim Miles**: Investigation (supporting). **Margaret Veitch**: Data curation (supporting); investigation (supporting); writing—review and editing (supporting). **Tamara Thomas**: Investigation (supporting). **Roland Ruscher**: Investigation (supporting); writing—review and editing (supporting). **Phurpa Wangchuk**: Investigation (supporting). **Paul Giacomin**: Conceptualization (supporting); data curation (supporting); formal analysis (supporting); investigation (supporting); methodology (supporting); validation (supporting); writing—review and editing (supporting). **Alex Loukas**: Conceptualization (supporting); funding acquisition (supporting); project administration (supporting); resources (equal); supervision (supporting); writing—review and editing (supporting). **Peter Nejsum**: Conceptualization (lead); funding acquisition (lead); project administration (lead); resources (lead); supervision (lead); writing—review and editing (equal).

## CONFLICT OF INTEREST STATEMENT

The authors declare no conflicts of interest.

## Supporting information



Supporting Information
