# Supplementary material for: Helminth extracellular vesicles co‐opt host monocytes to drive T cell anergy
Source: J Extracell Vesicles. 2025 Jan 16;14(1):e70027. doi: 10.1002/jev2.70027 (PMC11735955; doi:10.1002/jev2.70027)
Supplement: Supplementary file 1 — Supporting Information [file JEV2-14-e70027-s001.docx]

**Supplementary Information (SI)**

Supplementary Figure 1 (Fig. S1).


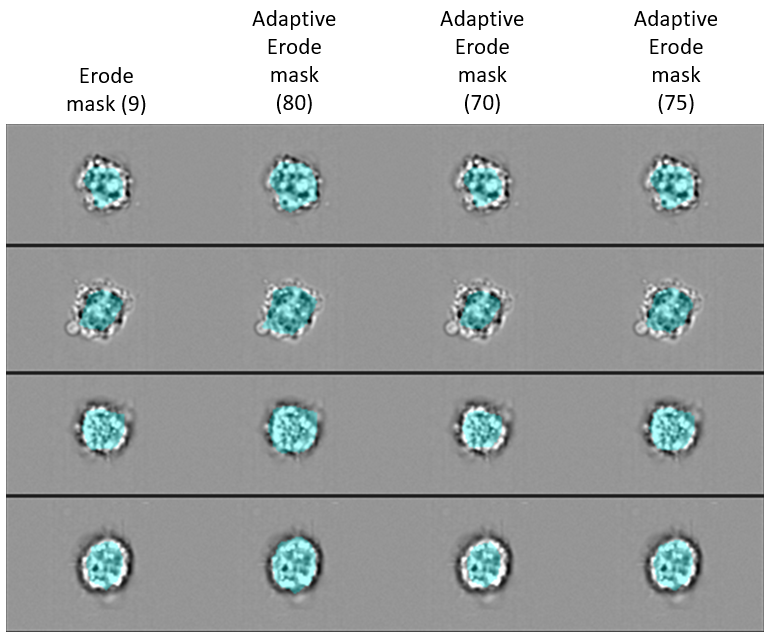


Fig. S1 Caption: Different masks, based on the brightfield image in channel 1, were created in the IDEAS software. The purpose was to find a mask covering the complete interior of the cell. The ‘AdaptiveErode mask (75)’ was chosen for internalization calculations.

Supplementary Figure 2 (Fig. S2)


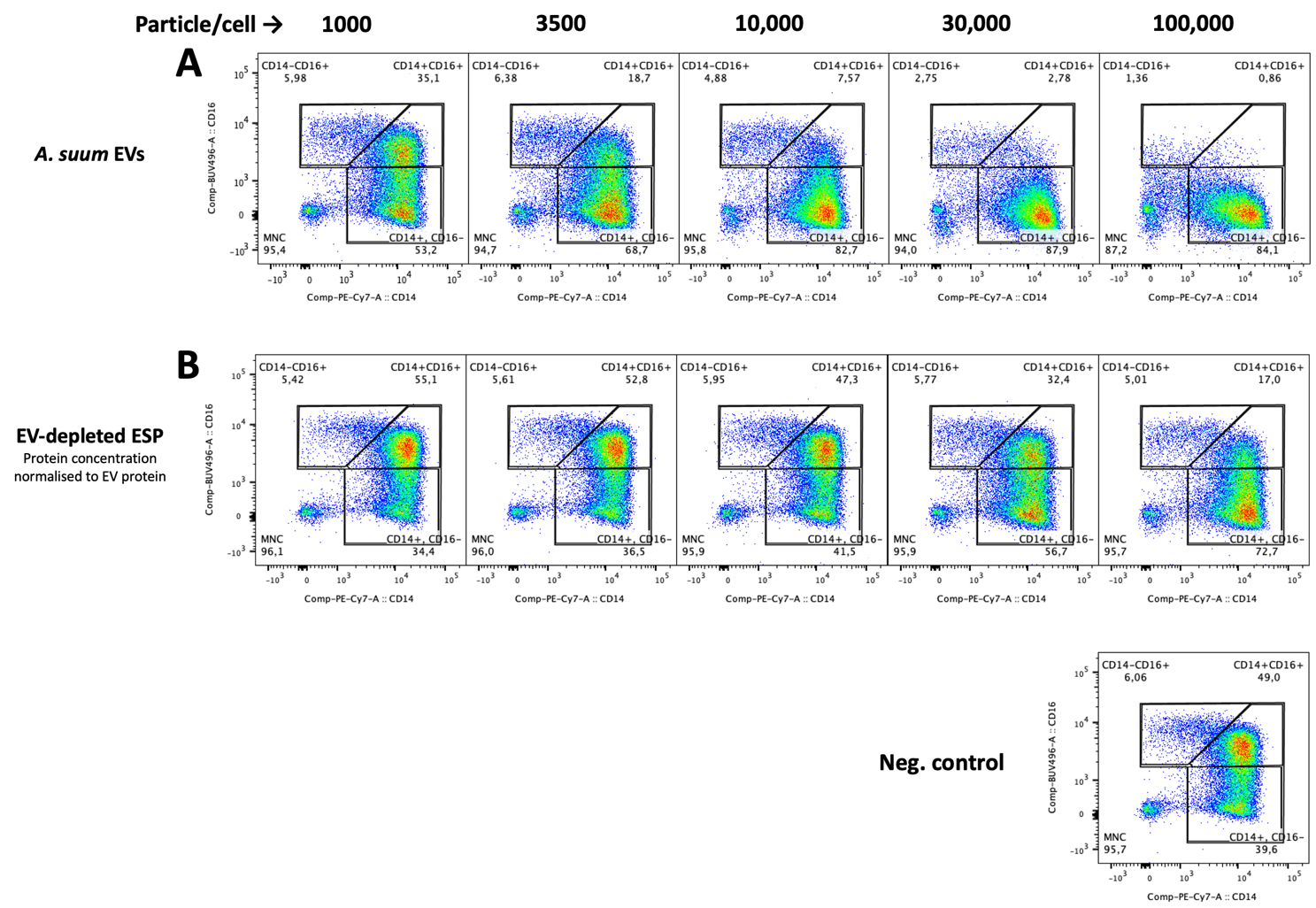


Fig. S2 Caption: FlowJo Scatterplots for comparison of monocyte subpopulations between stimulations with (A) increasing number of EV particles/cell and (B) corresponding EV-depleted ESP samples. Gating for the main population of monocytes (all MNC) and the three sub-populations of monocytes (classical, non-classical and intermediate monocytes) are present in both the test group and the negative control sample.

Supplementary Figure 3 (Fig. S3)


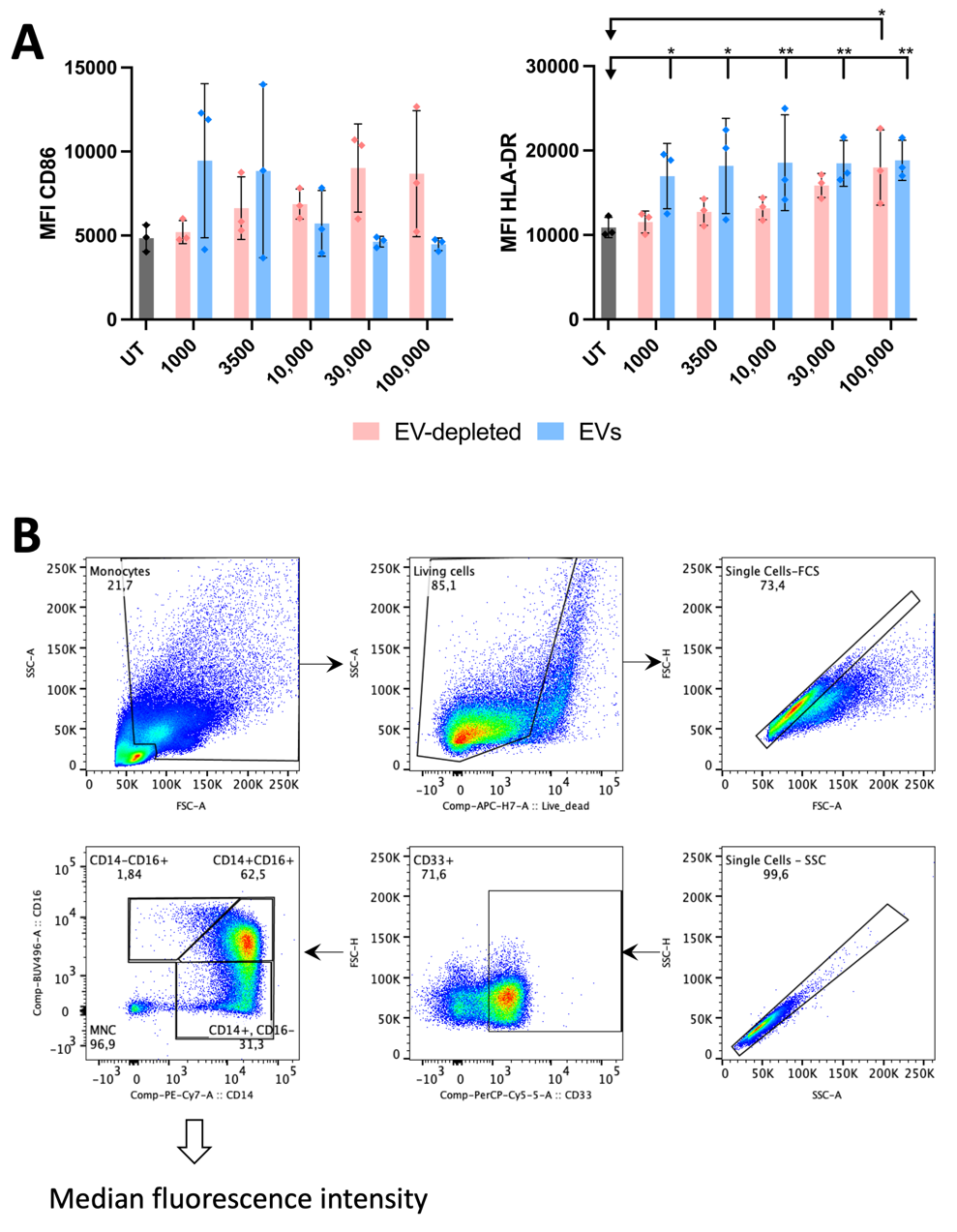


Fig. S3 caption: A) The median fluorescence intensity (MFI) of CD86 and HLA-DR expression on all MNCs was analysed by flow cytometry on human PBMCs stimulated with either EVs or EV-depleted ESP. The groups were compared using two-way ANOVA followed by a Tukey test. *p<0.05, **p<0.01, ***p<0.001, ****p < 0.0001. No significance between EVs and EC-depleted samples. Error bars: Mean ± SD. n = 3 donors. B) Sequential gating steps for MNCs, CD14^+^CD16^-^, CD14^+^CD16^+^ and CD14^-^CD16^-^ cell populations for the median fluorescence intensity measurements.

Supplementary Figure 4 (Fig. S4)


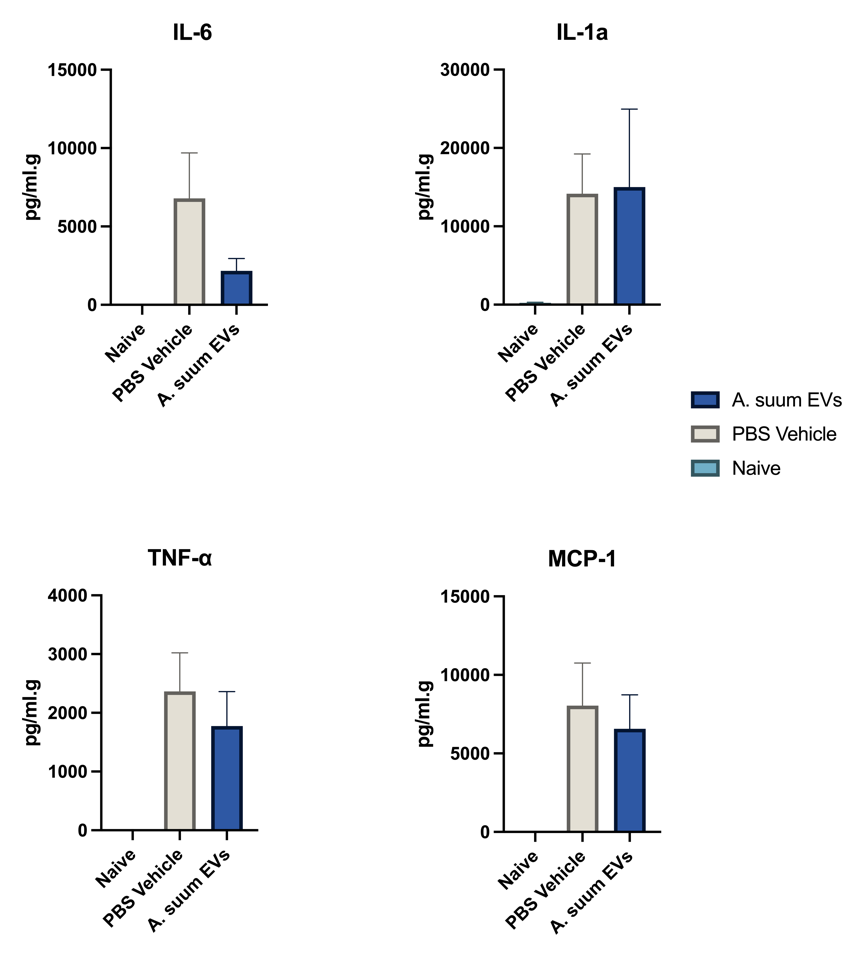


Fig. S4 Caption: Treatment of mice with *Ascaris suum* EVs results in a non-significant decrease in levels of IL-6 in the colon tissues of mice exposed to DSS. The concentration (pg/ml per gram of tissue) of the four detectable inflammatory cytokines is presented for naïve, PBS vehicle and *A. suum* EV-treated groups in the DSS mouse study.

Supplementary Figure 5 (Fig. S5)


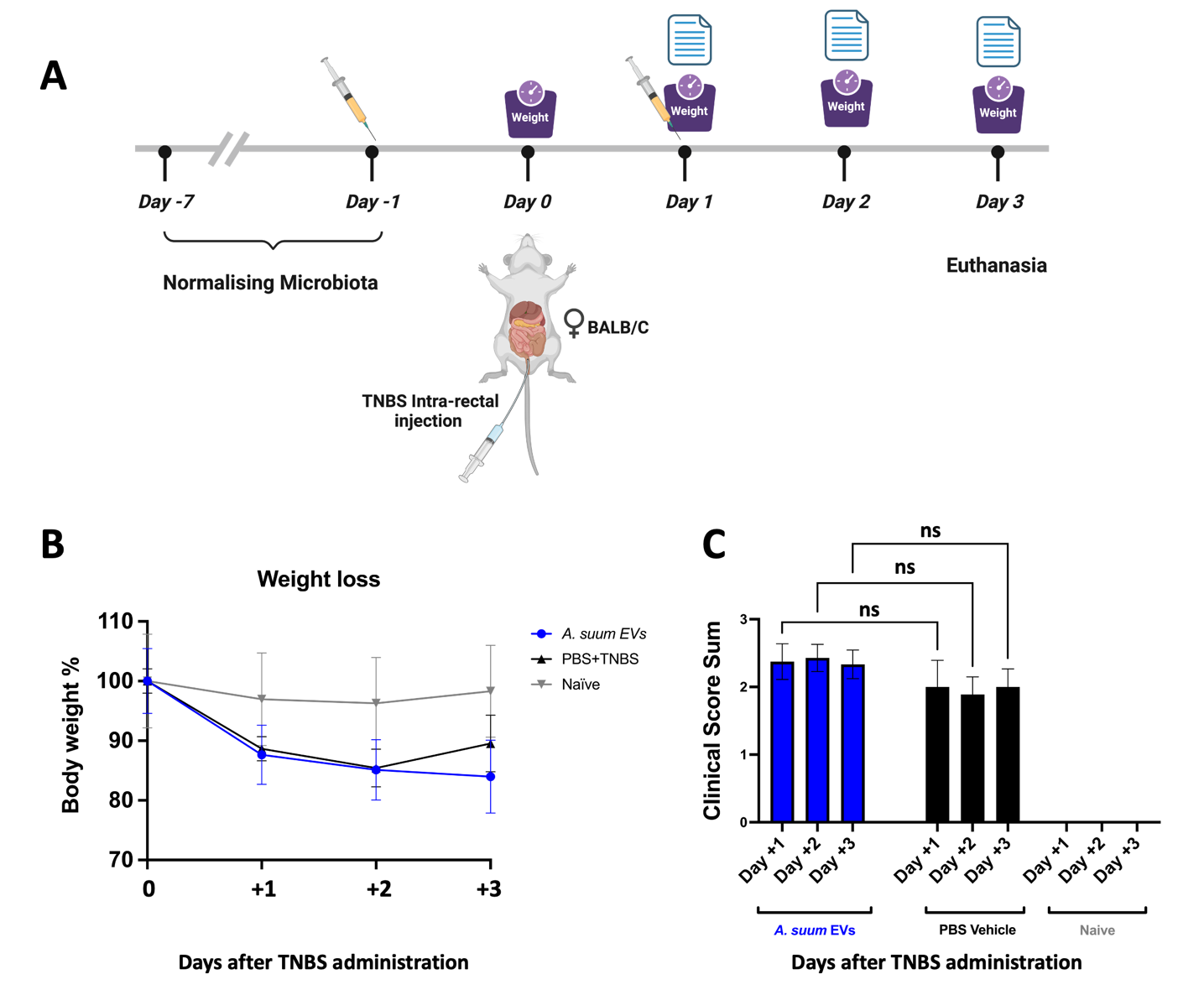


Fig S5 Caption: *Ascaris suum* EVs did not show protective effects on colitis in mice induced by TNBS.

**A.** Colitis was induced in female BALB/C mice intra-rectally, injecting 2.5 mg TNBS in 50% EtOH on day 0. A day prior to the colitis induction (day −1) and a day after TNBS induction (day +1), the mice in the treatment group received 20 µg *A. suum EVs*. Body weight and clinical scores were collected during the study.

**B.** Relative weight gain/loss for the studied mice during the study.

**C.** Cumulative clinical scores encompassing piloerection, faeces consistency, and rectal thickening/injury on days 1, 2 and 3 of the study. Statistical comparisons between the treatment and PBS vehicle groups were conducted using the Kruskal-Wallis test.

Supplementary Figure 6 (Fig. S6)


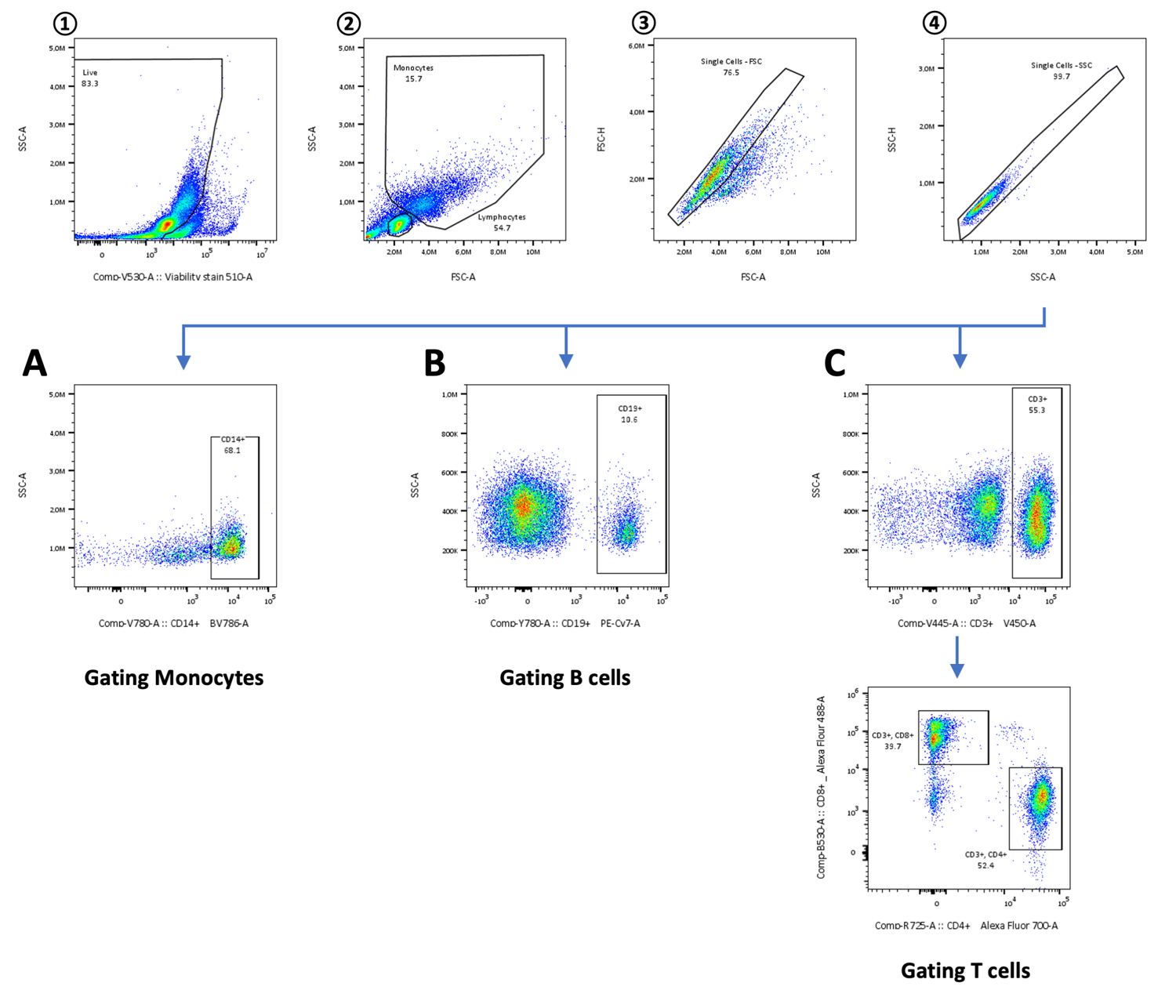


Fig. S6 Caption: Sequential gating steps were employed to identify monocytes, B and T cell populations within human peripheral blood mononuclear cells (PBMCs) during the extracellular vesicle (EV) uptake study. Within the single cell population, step 4, the monocyte (A), B cell (B) and T cell (C) populations were gated as CD14^+^, CD19^+,^ or CD3^+^, respectively.

Supplementary Table 1, List of all fluorochrome-conjugated antibodies used for flow cytometry in this study

| **Antibodies** | **Fluorochrome** | **Clone** | **Titer volume (µl)** | **Source** |
| --- | --- | --- | --- | --- |
| Anti-CD3 | V450 | UCHT-1 | 0.5 | BD Bioscience |
| Anti-CD4 | Alexa Flour 700 | SK3 | 0.19 | BD Bioscience |
| Anti-CD8 | Alexa Flour 488 | RPA-T8 | 0.06 | BD Bioscience |
| Anti-CD14 | BV786 | M5E2 | 0.5 | BD Bioscience |
| Anti-CD19 | PE-Cy7 | HIB19 | 0.06 | BD Bioscience |
| Fixable Viability Stain 510 |  |  | 1 | BD Bioscience |
| Anti-CD14 | APC | 61D3 | 5 | Invitrogen |
| Anti-CD86 | BUV395 | 2331 | 1.25 | BD Bioscience |
| Anti-CD16 | BUV496 | 3G6 | 2.5 | BD Bioscience |
| Anti-CD169 | BV421 | 7-239 | 0.65 | Biolegend |
| Anti-PD-L1 | BV605 | MIH1 | 1.25 | BD Bioscience |
| Anti-CD9 | BV786 | ML13 | 2.5 | BD Bioscience |
| Anti-CD33 | PerCP-Cy5.5 | WM53 | 1.25 | Biolegend |
| Anti-HLA-DR | PE | G46-6 | 5 | BD Bioscience |
| Anti-CD14 | PE-Cy7 | M5E2 | 1.25 | BD Bioscience |
| Anti-CCR2 | APC | K036C2 | 1.25 | Biolegend |
| Anti-CD47 | Alexa Flour 700 | CC2C6 | 5 | Biolegend |
| Fixable Near-IR |  |  | 1 | Invitrogen |
| Zombie Violet Viability Dye |  |  | 1:1000 | Biolegend |

Supplementary Figure 7 (Fig. S7)


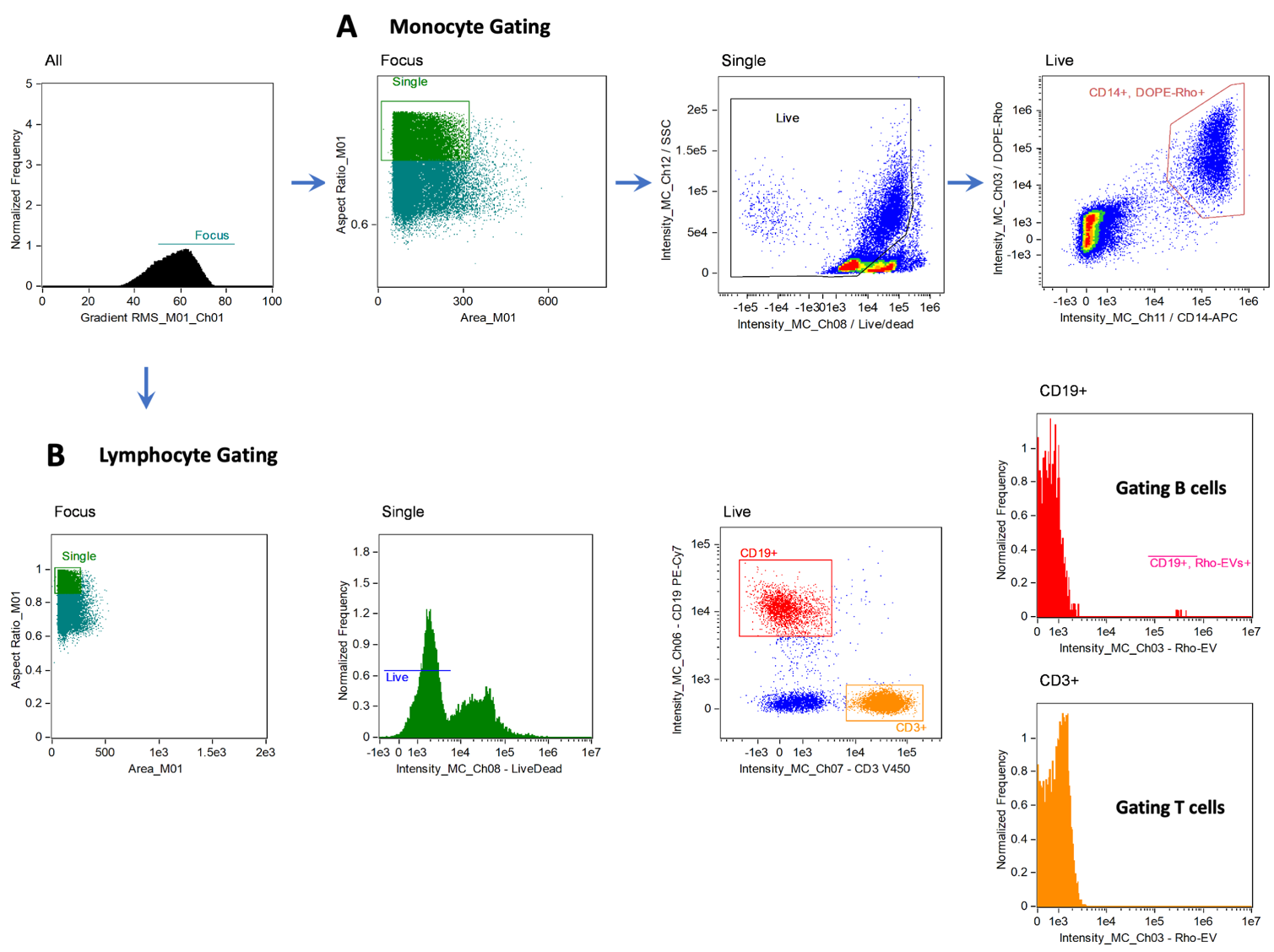


Fig. S7 Caption: Sequential gating steps from IDEAS employed to data from the ImageStream^X^ Mk II identify monocytes (A); and lymphocytes (B) within human peripheral blood mononuclear cells (PBMCs) for the extracellular vesicle (EV) uptake study. Cells were gated first for the brightfield image to be in focus, single cells, live cells and finally CD14/DOPE-Rho double-positive cells were evaluated for internalization. The percentage of B-cells (CD19^+^) and T-cells (CD3^+^) being positive for DOPE-Rho was 0.2 and 0.23, respectively.

Supplementary Figure 8 (Fig. S8)


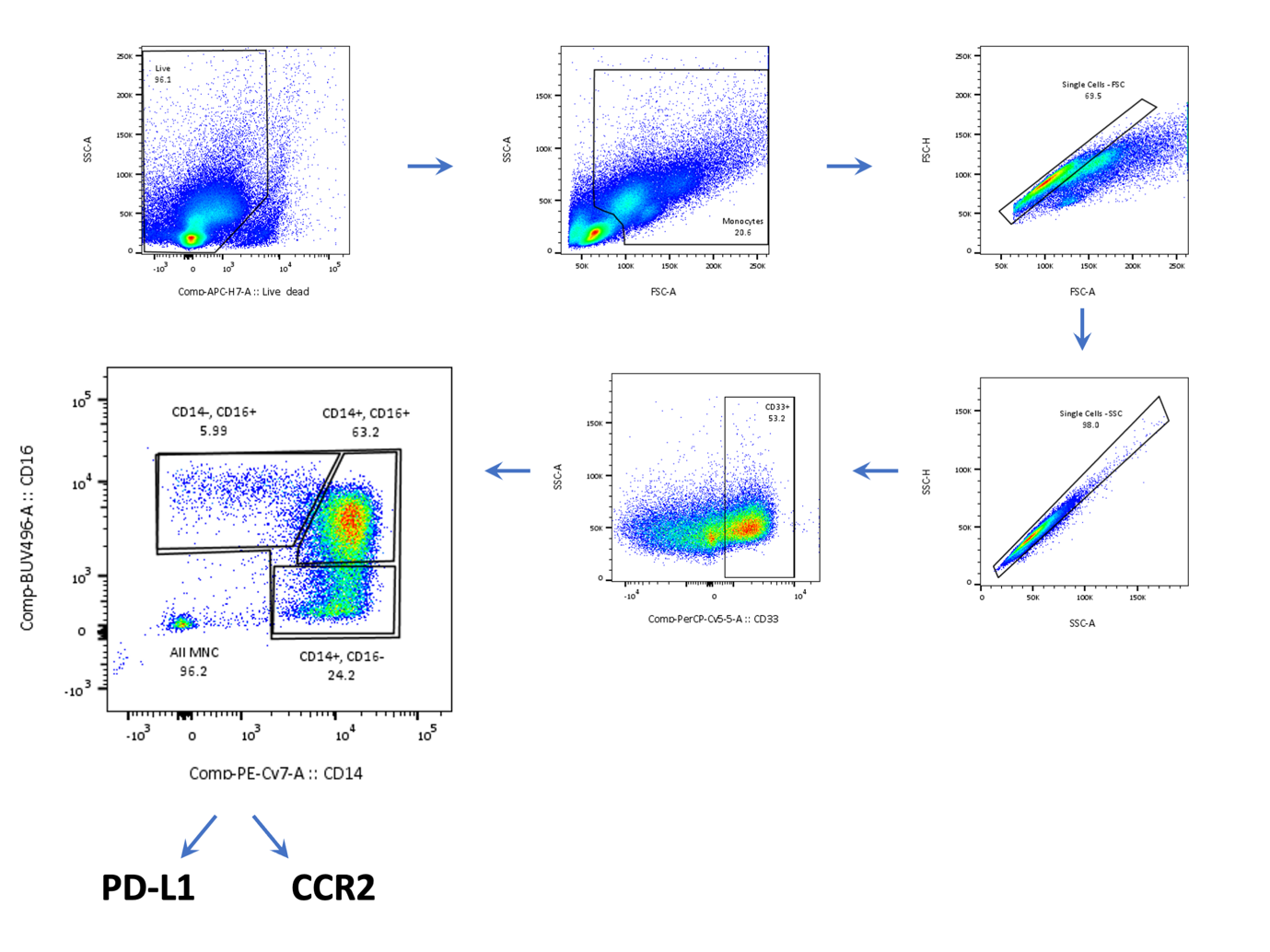


Fig. S8 Caption: Sequential gating steps employed to identify monocyte population and their death-ligand 1 (PD-L1) and CC chemokine receptor 2 (CCR2) profiling within human peripheral blood mononuclear cells (PBMCs) for the PNGase treatment experiment.

Supplementary Figure 9 (Fig. S9)


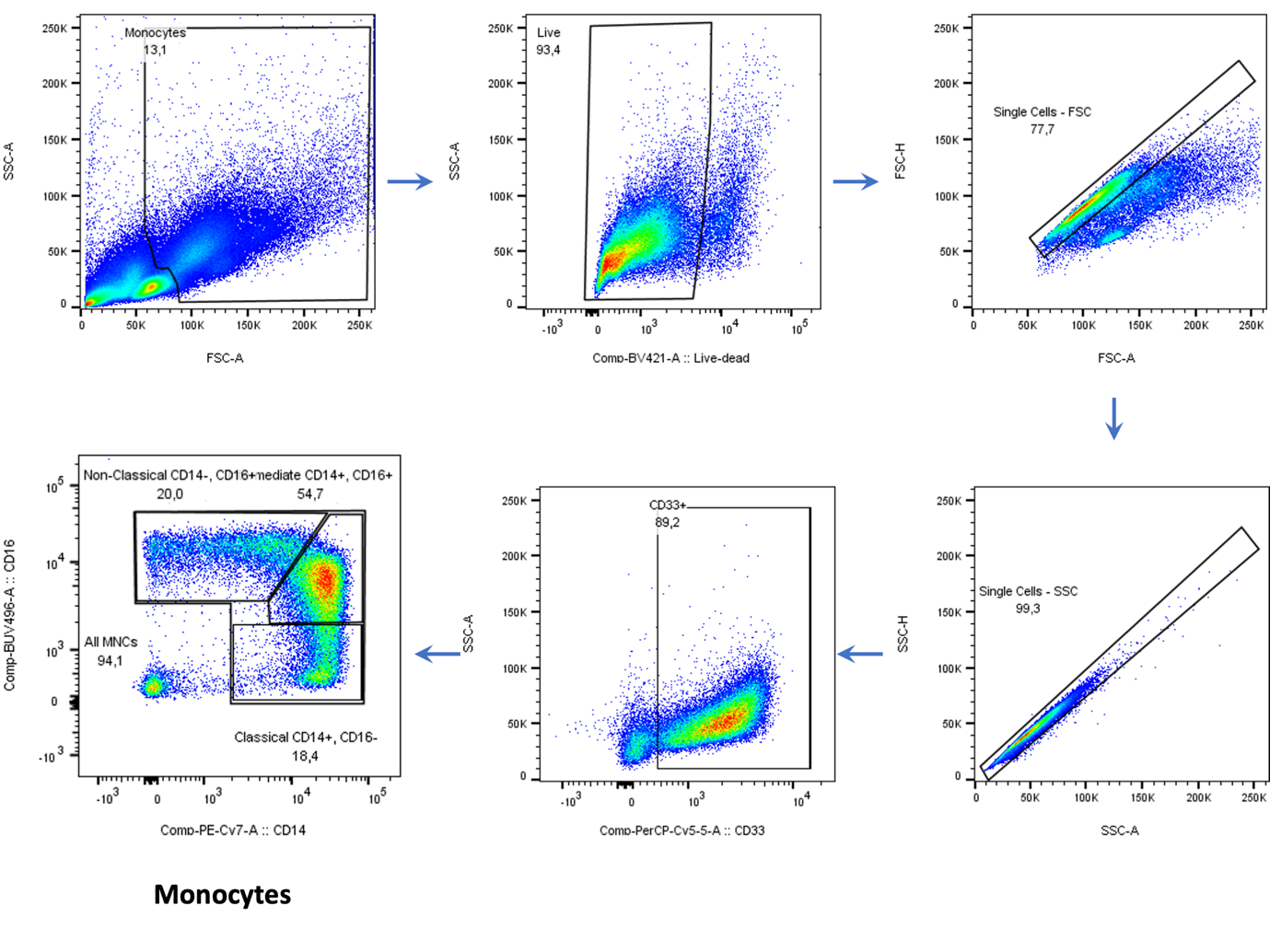


Fig. S9 Caption: Sequential gating steps employed to identify non-classical, intermediate, and classical monocyte populations within human peripheral blood mononuclear cells (PBMCs) for the inhibition of CD16 and CCR2 cleavage experiment.

Supplementary Figure 10 (Fig. S10)


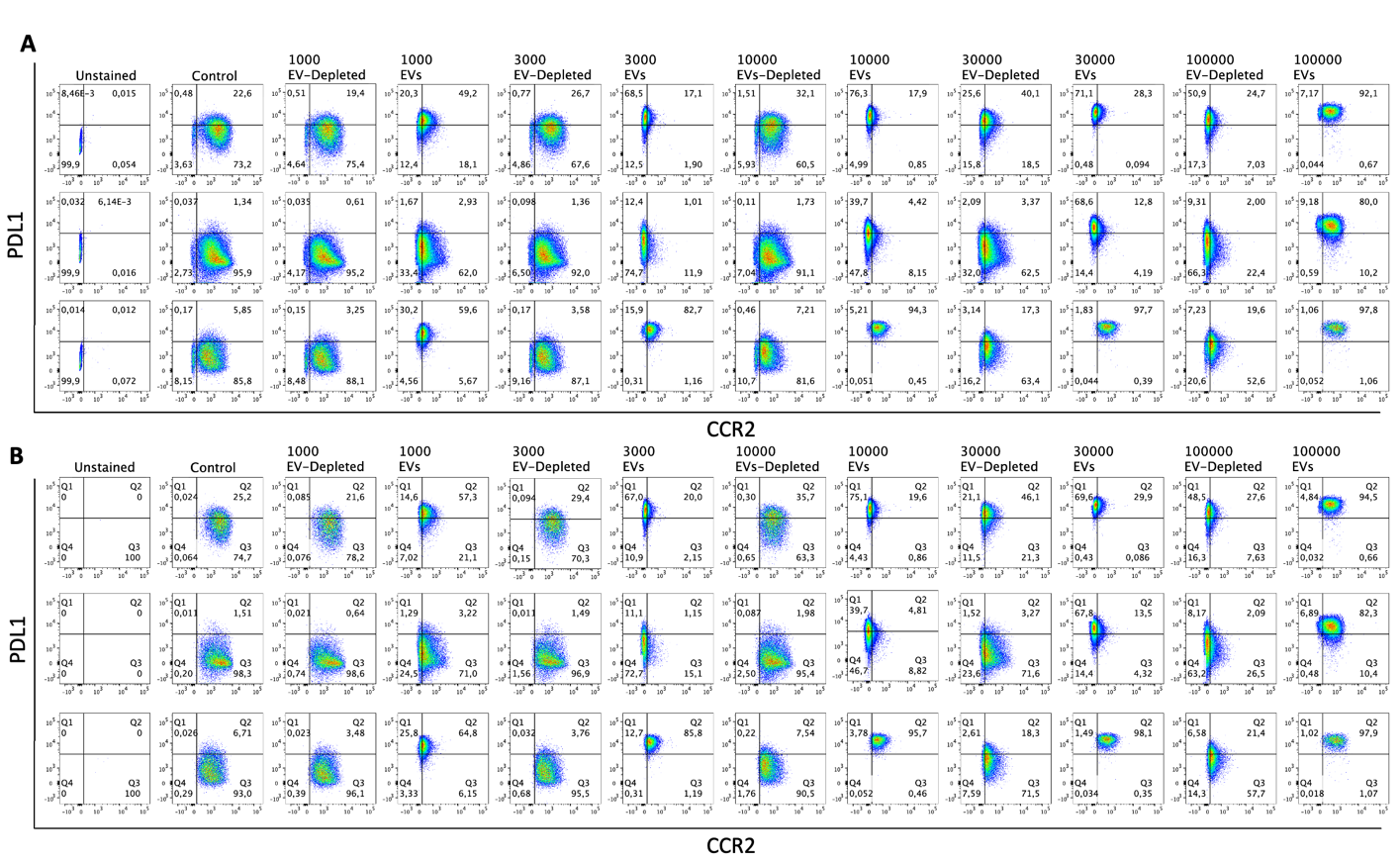


Fig. S10 Caption: Scatterplots and gating strategy concerning the expression of PD-L1 and CCR2 and the composition of monocyte subpopulations in the presence and absence of *A. suum* EVs. A) All monocyte populations exposed to different concentrations of *A. suum* EVs. B) CD14^+^CD16^-^ Cells subpopulation.

Supplementary Figure 11 (Fig. S11)


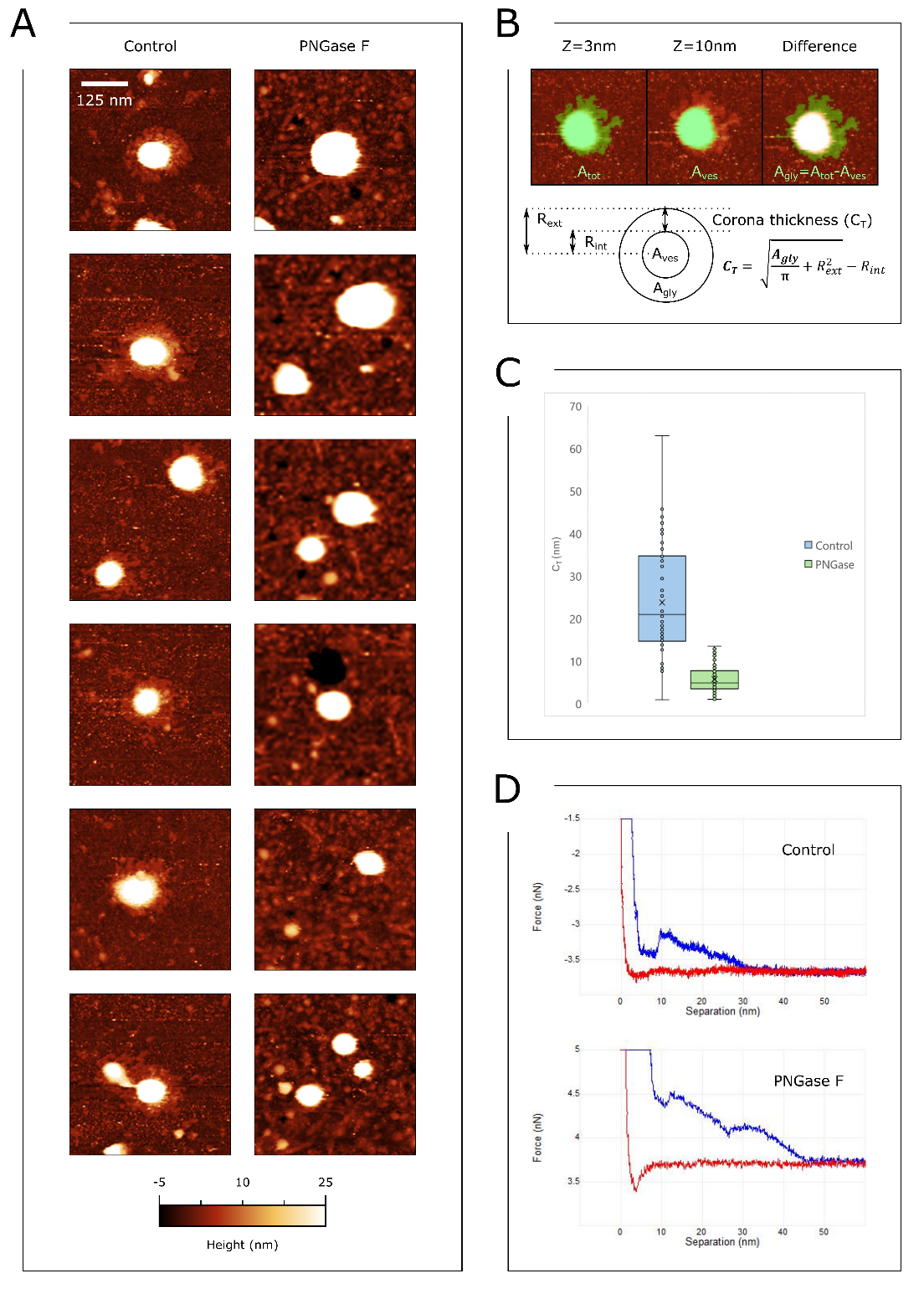


Fig. S11 caption: Removal of the glycan corona via peptide:N-glycosidase F (PNGase F) treatment as observed via AFM.

A. Representative AFM micrographs of individual untreated (left column) and 400 U/ml PNGase F‑treated (right column) *A. suum* EVs. All images share the same Z scale and lateral dimensions.

B. The method used to quantify the amount of glycan corona on individual EVs via AFM morphometry. The total projected area of an EV (A_tot_) is first measured by applying a threshold at Z=3nm above the average background value. The same is then done at Z=10nm to measure the area projected by the bilayer-enclosed portion of the EV (A_ves_). The glycan corona area (A_gly_) is then calculated by subtracting A_tot_ - A_ves_. Finally, the corona is modeled for each individual EV as an annulus of area A_gly_ surrounding a disk of area A_ves_; the calculated annulus radius C_T_ is employed to quantify the degree of glycan decoration.

C. Box plot of the glycan corona thickness C_T_ before (control) and after PNGase F treatment. C_T_ drops from 24±13nm (N=48) to 6±3nm (N=243), attesting a significant glycan removal.

D. Representative force/distance plots of nanoindentations performed on individual untreated (control) and PNGase-treated EVs. In both cases, the characteristic linear force regime of intact EVs [Vorselen et al, Front. Mol. Biosci. 2020, <https://doi.org/10.3389/fmolb.2020.00139is>] is observed, confirming that extensive glycan removal does not compromise the structural integrity of the EVs’ bilayer.
